# Functional requirements of protein kinases and phosphatases in the development of the *Drosophila melanogaster* wing

**DOI:** 10.1093/g3journal/jkab348

**Published:** 2021-10-02

**Authors:** Cristina M Ostalé, Nuria Esteban, Ana López-Varea, Jose F de Celis

**Affiliations:** Centro de Biología Molecular “Severo Ochoa,” CSIC and Universidad Autónoma de Madrid, Madrid 28049, Spain

**Keywords:** phosphorylation, wing morphogenesis, genetic screen, RNAi

## Abstract

Protein kinases and phosphatases constitute a large family of conserved enzymes that control a variety of biological processes by regulating the phosphorylation state of target proteins. They play fundamental regulatory roles during cell cycle progression and signaling, among other key aspects of multicellular development. The complement of protein kinases and phosphatases includes approximately 326 members in *Drosophila*, and they have been the subject of several functional screens searching for novel components of signaling pathways and regulators of cell division and survival. These approaches have been carried out mostly in cell cultures using RNA interference to evaluate the contribution of each protein in different functional assays and have contributed significantly to assign specific roles to the corresponding genes. In this work, we describe the results of an evaluation of the *Drosophila* complement of kinases and phosphatases using the wing as a system to identify their functional requirements *in vivo*. We also describe the results of several modifying screens aiming to identify among the set of protein kinases and phosphatases additional components or regulators of the activities of the epidermal growth factor and insulin receptors signaling pathways.

## Introduction

Reversible protein phosphorylation was first described in the 1950s (Krebs and Fischer 1955) and since then many studies have emphasized that phosphorylation is one of the main regulatory mechanisms modifying protein activity and consequently a variety of cellular behaviors including cell cycle progression, cell death, metabolism, tissue homeostasis, cell motility, and cell differentiation (Cohen 2001). The phosphorylation state of a protein is a determinant of its biochemical activity and defines protein stability and subcellular location. Protein phosphorylation also allows transitions between active and inactive conformations and influences the repertoire of interactions with other proteins. Not surprisingly, several diseases such as obesity, cancer, and inflammation are related with aberrant phosphorylation, emphasizing its essential role in the regulation of cellular biology (reviewed in Shchemelinin *et al.* 2006; Tonks 2006; Hendriks *et al.* 2013).

The phosphorylation/dephosphorylation of proteins is mediated by protein kinases and protein phosphatases, enzymes that catalyze the transfer of phosphate groups to or from its targets, respectively (Hunter 1995; Shchemelinin *et al.* 2006; Hendriks *et al.* 2013). Kinases represent one of the largest protein families encoded in eukaryotic genomes, accounting for around 500 genes in humans and 328 genes in *Drosophila melanogaster* (Morrison *et al.* 2000). Phosphatases constitute a smaller group, including about 200 and 192 genes in humans and fly, respectively (Morrison *et al.* 2000). There are no *Drosophila*-specific families of kinases or phosphatases, and each subfamily presents small complexity and low redundancy (Manning *et al.* 2002). These characteristics, and the facility of genetic manipulation in this organism, make *Drosophila* a suitable model for the functional study of these gene families in developing tissues and cell cultures (Mattila *et al.* 2008; Read *et al.* 2013; Swarup *et al.* 2015). One organ that is particularly well suited for such functional approaches is the wing, a flat structure of epidermal origin that has been systematically used as a model system to dissect the molecular components and cell biology underlying epithelial development (Molnar *et al.* 2011; Hariharan 2015). 

The *Drosophila* wing is a cuticular structure resulting from the differentiation of an epidermal tissue named wing imaginal disc. All features decorating the wing such as sensory organs, pigmentation, and veins are the results of the differentiation, during pupal development, of epidermal cells that were genetically specified during the growth of the wing imaginal disc (Ostalé *et al.* 2018). In this manner, wing patterning, as well as its size and shape, is determined during the development of the wing disc. There are multiple cellular processes impinging on wing development that are regulated by the opposing actions of kinases and phosphatases on their targets. These processes include cell growth and division, the acquisition and maintenance of apical-basal and planar polarities and vein differentiation among others (Bettencourt-Dias *et al.* 2004; Chen *et al.* 2007; Read *et al.* 2013; Parsons *et al.* 2017). In addition, protein phosphorylation pervades as a regulatory mechanism in multiple signal transduction pathways regulating pattern formation and cell differentiation.

One significant advantage of the wing for genetic analysis is the variety and specificity of phenotypic responses to genetic perturbations. For example, altering the activity of signaling pathways results in precise and pathway-specific phenotypes affecting the size and shape of the wing, the formation and polarity of the trichomes differentiated by each epithelial cell, and the position and differentiation of veins (Molnar *et al.* 2011; Ostalé *et al.* 2018). These phenotypes allow the grouping of novel mutations or knockdown conditions and can be used as a first approximation to assign gene functions by phenotypic comparison. An additional advantage of the wing for genetic analysis is the possibility of carrying out “modifier” screenings using sensitized backgrounds in which the activity of a given signaling pathway is altered. It is expected that sensitized genetic backgrounds help to identify additional components of these pathways or other molecular elements affecting their activities. For example, modifying screens have been instrumental in identifying components of the EGFR and Wnt pathways during imaginal development (Friedman and Perrimon 2006; McElwain *et al.* 2011; Swarup *et al.* 2015).

In this work, we describe the adult wing phenotypes resulting from the individual knockdown of most annotated *Drosophila* kinases and phosphatases, with particular emphasis in protein kinases and phosphatases. We find that 53% of protein kinases and 40% of protein phosphatases result in mutant wing phenotypes affecting the size, pattern, and differentiation of this organ. This percentage is higher compared to the percentage found for Carbohydrate, Lipid, and Nucleoside kinases (101 genes; 29% knockdowns with a phenotype). In addition, we have constructed and used sensitized genetic backgrounds in which the activities of the epidermal growth factor receptor (EGFR) and insulin receptor (InR) pathways are altered to screen the same collection of protein kinases and phosphatases for genetic interactions.

## Materials and methods

### *Drosophila* stocks and genetics

We used the *Gal4* lines *sal^EPv^-Gal4* and *nub-Gal4.* The expression of *sal^EPv^-Gal4* is restricted to the wing blade territory located between the vein L2 and the intervein L4/L5 (Cruz *et al.* 2009). The expression of *nub-Gal4* is generalized in the entire wing pouch and hinge. For the modifier screens, we used the *UAS* lines *UAS-GFP*, *UAS-dicer2*, *UAS-InR^DN^* (*P{UAS-InR.K1409A}*; BSCD8252), *UAS-InR^Act^* (*P{UAS-InR.R418P}*; BSCD8250), *UAS-ERK^sem^* (Brunner *et al.* 1994), *UAS-ERK-RNAi* (VDCR 109108), *UAS-EGFR^λtop^* (BDSC59843), and *UAS-EGFR-RNAi* (VDCR 107130). These lines were combined or recombined with *sal^EPv^-Gal4*. Virgin females of *sal^EPv^-Gal4 UAS-GFP/CyO*, *sal^EPv^-Gal4/CyO; UAS-InR^DN^/TM6b, sal^EPv^-Gal4 UAS-InR^Act^/CyO, sal^EPv^-Gal4/CyO; UAS-ERK^sem^/TM6b, sal^EPv^-Gal4 UAS-ERK-RNAi/CyO*, UAS-EGFR^λtop^*; sal^EPv^-Gal4/CyO*, and *sal^EPv^-Gal4/CyO; UAS-EGFR-RNAi/TM6b* were crossed with males from the collection of *UAS-RNAi* of the complement of protein kinases and phosphatases. The *UAS-RNAi* lines used for kinases and phosphatases are listed in Supplementary Table S1. Most *UAS-RNAi* strains were obtained from the Vienna *Drosophila* RNAi Center (VDCR; 478 strains), and some from the Bloomington Stock Center (BDSC; 7 strains), and the National Institute of Genetics (NIG-FLY; 6 strains). The knockdown phenotypes of these genes were determined in *UAS-dicer2/+; nub-Gal4/UAS-RNAi and UAS-dicer2/+; sal^EPv^-Gal4/UAS-RNAi* combinations. We aimed to describe each mutant wing using a simplified nomenclature summarizing the main components of its phenotype. Many combinations displayed late larval (LL) or pupal lethality (PL). In many cases, dead pupae observed in the puparium showed necrotic patches in the position normally occupied by the wings (nec). Flies showing a total failure in the formation of the wings were named “nW” (no-wing). Wings showing wing size changes were defined as “S” (wing size smaller than normal) and “S(L)” (wing size larger than normal). When changes in size were accompanied by changes in the pattern of veins, the phenotype was named “S-P.” Changes affecting primarily the wing veins were defined as V− (loss of veins) and V+ (excess of veins). All defects related to the wing margin consisting in the loss of wing margin stretches were defined as “WM.” Defects in the apposition of the dorsal and ventral wing surfaces, observed in the form of blisters, were considered as failures in dorsoventral adhesion, and were named “WA.” Similarly, defects in the global shape of the wing were defined as wing shape (“WS”), and they include lanceolate wings (lan) and dumpy wings (dp). In some cases, the wing cuticle appeared with an abnormal general appearance, brighter than normal, not entirely unfolded or with necrotic patches. These wings were classified as wing differentiation defects (“WD”). In other cases, wing cuticle was darker than normal, and these cases were named “WP” (wing pigmentation defects). Changes in the number of trichomes formed by each cell, which normally differentiate only one trichome, as well as alterations in trichome polarity and spacing, were defined as alterations in cell differentiation (“CD”). A very frequent phenotype observed in combinations between *nub-Gal4 and UAS-RNAi* strains of the KK VDCR collection result in the formation of adults with the wings totally folded (“WF”). This phenotype is a consequence of a UAS insertion affecting the gene *tiptop* (Green *et al.* 2014; Vissers *et al.* 2016). As discussed elsewhere (López-Varea *et al.* 2021), the same KK UAS-RNAi lines in combination with the driver *sal^EPv^-Gal4* result in the formation of normal wings, and consequently, all WF wings where we could not observe any other phenotype were considered as wild type for all quantifications. Finally, we included the bins “strong” (s) and weak (w) in the phenotypic description, to give an indication of relative phenotypic strength. Unless otherwise stated, crosses were done at 25°C.

We did not measure the efficiency of mRNA knockdown in these genetic combinations. It was estimated in a collection 64 *UAS-RNAi/act-Gal4* viable combinations that the reduction in mRNA levels varies from 95% to 10%, and that an estimated 15–40% of *UAS-RNAi* insertions are inactive (Dietzl *et al.* 2007; Perkins *et al.* 2015). For these reasons, a fraction of combinations without a mutant phenotype could be due to insufficient knockdown efficiency. In addition, we generally used only one *UAS-RNAi* strain per gene. However, from our data (López-Varea *et al.* 2021, *G3* submitted), we know that lines targeting the same gene result in similar qualitative phenotypes (202 out of 281 cases analyzed; see López-Varea *et al.* 2021) and that in the remaining cases (82% of 79 genes), the more frequent situation is that one *nub-Gal4/UAS-RNAi* combination results in a mutant phenotype and the other in wild-type flies, again pointing to different knockdown efficiencies between independent strains. In agreement, when we compared our results with a previous RNAi screen of *Drosophila* protein kinases and phosphatases that used multiple *UAS-RNAi* lines to target each gene (Swarup *et al.* 2015), we found a coincidence for genes showing a wing phenotype in 82% of the genes we identified. The remaining 18% of genes correspond to cases described in Swarup *et al.* (2015) as “mutant wing” where we could not detect a mutant phenotype. These genes are indicated in red lettering in Supplementary Table S1.

### Wing and disc measurements

Wing pictures were made with a Spot digital camera coupled to a Zeiss Axioplan microscope, using the 5X and 40X objectives for wings and for wing regions, respectively. Cell size was estimated from the number of trichomes in a dorsal region located between the L2 and L3 longitudinal veins. The number of cells was calculated using cell density and wing size values.

### Immunohistochemistry

We used the rabbit antibodies anti-phospho-Histone3 and anti-cleaved Cas3 (Cell Signaling Technology). Alexa Fluor secondary antibodies (used at 1:200 dilution) were from Invitrogen. To stain the nuclei we used TO-PRO-3 (Invitrogen). Imaginal wing discs were dissected, fixed, and stained as described in de Celis (1997). Confocal images were taken in an LSM510 confocal microscope (Zeiss). All images were processed with the program ImageJ 1.45 s (NIH, USA) and Adobe Photoshop CS3.

### Statistical analysis

All numerical data including wing size and cell size were collected and processed in Microsoft Excel (Microsoft Inc.). The data and ratios between number of cells were expressed as means + standard error of the mean (SEM) and were compared using a *T*-test. *P*-values were adjusted by false discovery rate method using R-studio platform. We consider a significant *P*-value lower than 0.05 (*), 0.01 (**) and 0.001 (***).

### Gene expression

We used RNA-Seq reads from run SRR3478156, corresponding to control larvae expressing Gal4/GFP data obtained from dissected wing imaginal discs (Flegel *et al.* 2016) and GeneChip™ *Drosophila* Genome 2.0 Affymetrix array data (Organista *et al.* 2015) to determine expression or not expression in the wing disc for all genes encoding kinases and phosphatases.

The authors affirm that all data necessary for confirming the conclusions of the article are present within the article, figures, tables, and Supplementary information.

## Results and discussion

### Phenotypic screen of kinases in the wing

**Figure 1:**
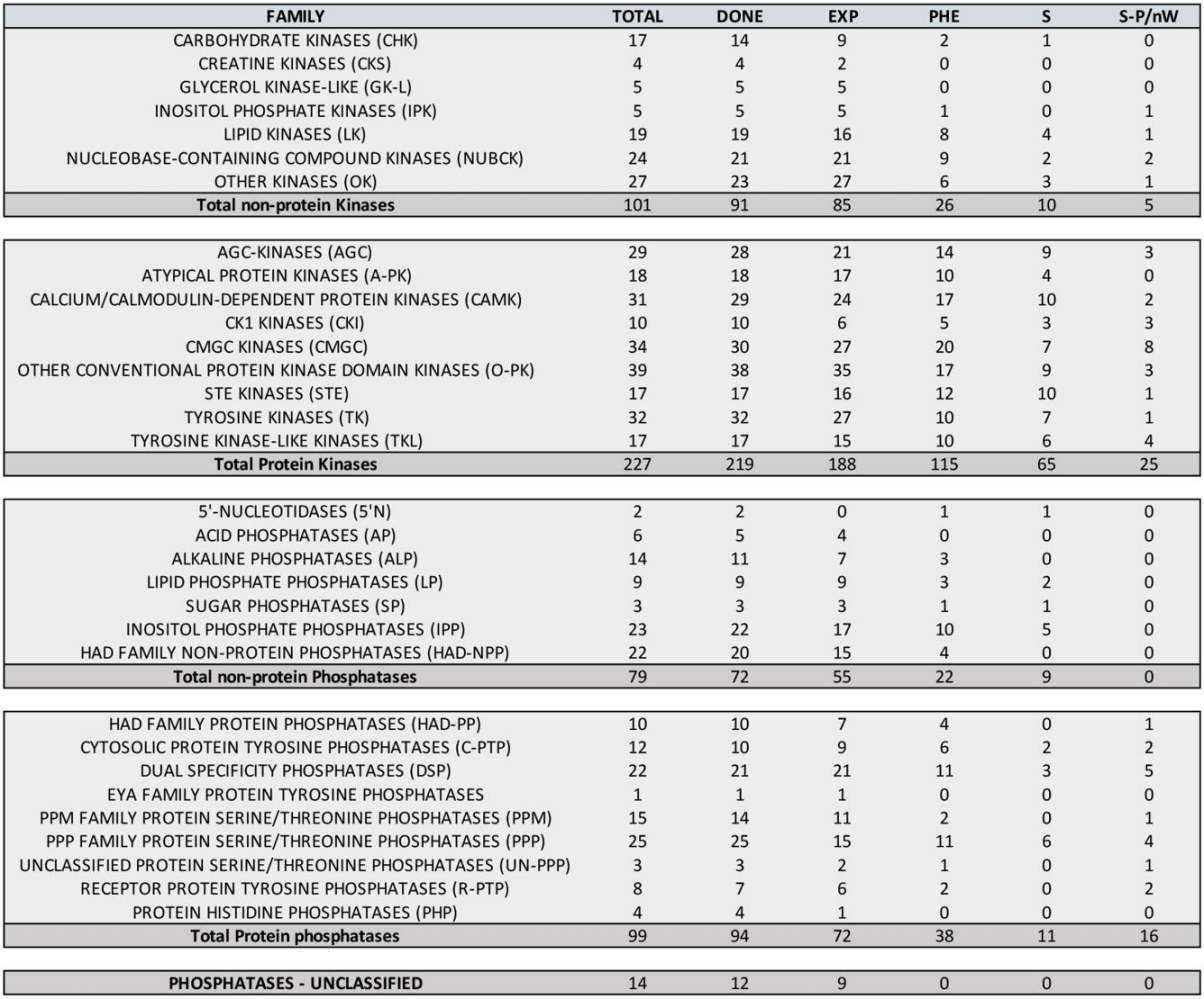
Global parameters of kinases and phosphatases expression and knockdown phenotypes. Summary of the number of genes (TOTAL), genes analyzed (DONE), genes expressed in the wing disc (EXP), genes with a knockdown wing phenotype (PHE), gene knockdowns causing altered wing size (S), and gene knockdowns causing loss of wing or strong defects in wing size and pattern phenotype (S-P/nW).

**Figure 2:**
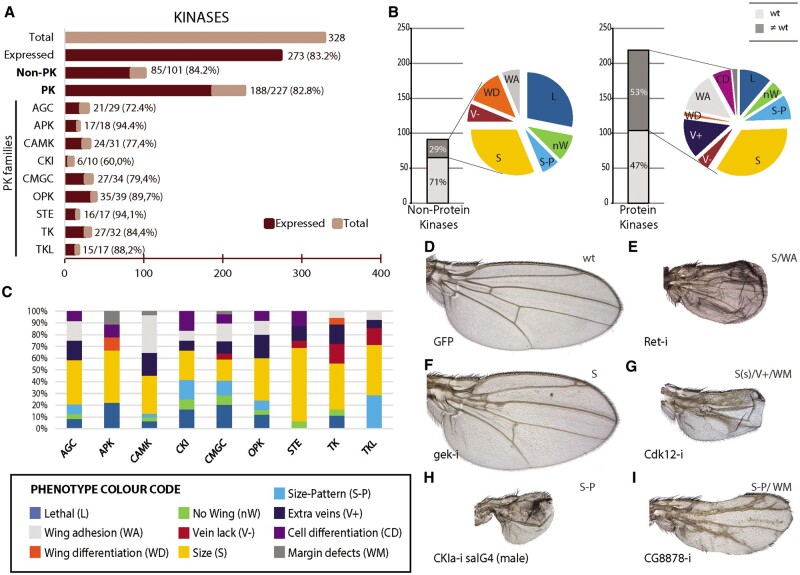
Global results of the RNAi screen for the complement of *Drosophila* kinases. (A) Fraction of kinases genes expressed from total (338 genes) and separated into the groups nonprotein kinases (Non-PK) and protein kinases (PK) of the classes AGC Kinases (AGC), Atypical protein kinases (APK), Calcium/Calmodulin-dependent protein kinases (CAMK), CK1 Kinases (CKI), CMGC Kinases (CMGC), other conventional protein kinase domains (OPK), Steryle20 kinases (STE), Tyrosine Kinases (TK), and Tyrosine kinase-like kinases (TLK). (B) Number of genes and percentages of genes with a knockdown mutant phenotype (dark gray sections of each column) or without mutant phenotype (light gray section of each column). Colored sectors show the percentage of each phenotype for nonprotein kinases (left) and for protein kinases (right). Lethality (L; dark blue sector), loss of wing (nW; green sector), changes in wing size and pattern (S-P; light blue sector), changes in size (S; yellow sector), loss of veins (V−; red sector), extra or thicker veins (V+; dark blue sector), wing differentiation defects (WD; orange sector), wing adhesion defects (WA; gray sector), and trichome differentiation or size defects (CD; purple sector). (C) Percentage of lethal and visible mutant phenotypes observed in the AGC, APK, CAMK, CKI, CMGC, OPK, STE, TK, and TKL classes using the same color code as above and indicated below the columns. (D–I) Representative examples of *UAS-Dicer2/+; nub-Gal4/UAS-GFP* (D), *UAS-Dicer2/+; nub-Gal4/UAS-Ret-RNAi* (E), *UAS-Dicer2/+; nub-Gal4/UAS-gek-RNAi* (F), *UAS-Dicer2/+; nub-Gal4/UAS-Cdk12-RNAi* (G), *UAS-Dicer2/+; nub-Gal4/UAS-CKIa-RNAi* (H) and *UAS-Dicer2/+; nub-Gal4/UAS-CG8878-RNAi* (I) adult wings. Defects in wing size (S), wing size and vein patterning (S-P), extra- or thicker veins (V+), defects in the wing margin (WM), and appearance of wing blisters (WA) are indicated in the upper-right corner of each picture.

Kinases catalyze the transfer of a phosphate group from ATP to a substrate molecule. To compile a list of kinases (and phosphatases, see below), we used the classification provided in the FlyBase gene group list (http://flybase.org/lists/FBgg/) and the annotation of protein kinases provided by Morrison *et al.* (2000). We included in our analysis carbohydrate, lipid, nucleoside, and protein kinases, resulting in a group of 328 genes (Figures  1 and 2A). As a general procedure for the screen, we used only one UAS-RNAi line per gene. We first crossed *UAS-RNAi* males (Supplementary Table S1) with *UAS-dicer2; nub-Gal4/CyO* females. In all cases, where the progeny *UAS-dicer2/+; nub-Gal4/UAS-RNAi* was lethal or resulted in flies with rudimentary wings (42 out of 310 crosses performed), we crossed the corresponding *UAS-RNAi* lines with *UAS-dicer2; sal^EPv^-Gal4/CyO* females. The *UAS-dicer2/+ sal^EPv^-Gal4/UAS-RNAi* combinations were always viable and were used to classify phenotypically the corresponding RNAi lines.

Carbohydrate, Lipid, and Nucleoside kinases include 101 proteins mostly involved in metabolic pathways (71%; Supplementary Table S1). The corresponding genes are generally expressed in the wing disc (84%; Figures  1 and 2A) and their knockdowns result in lethality or a wing phenotype in a low percentage of cases (29%; Figures  1 and 2B). The phenotypes most frequently observed after knockdown of nonprotein kinases consisted in a reduction of the size of the wing (S, 31%; Figures  1 and 2B) and defects in wing cuticle differentiation (WD, 13%; Figure  2B).

Protein kinases comprise a single protein superfamily having a common catalytic structure (Morrison *et al.* 2000). These enzymes are further subdivided into distinct groups based on their structural and functional properties (Hanks and Hunter 1995). Most of the 227 protein kinases genes are expressed in the wing disc (83%; Figures  1 and 2A) and in 53% of them we identified lethality or a mutant wing phenotype in *UAS-dicer2/+; nub-Gal4/UAS-RNAi* or *UAS-dicer2/+; sal^EPv^-Gal4/UAS-RNAi* combinations (Figures  1 and 2, B and C and Table  1). The most frequent alterations observed were changes in the size of the wing (S), in many cases accompanied by changes in the position (size and pattern; S-P) or the differentiation (size and vein formation; S/V) of the veins (Table  1; Figure  2, B and C). Other changes in wing morphology consist in blisters, caused by a failure in the adhesion between the dorsal and ventral wing surfaces (wing adhesion; WA), or failures in the formation of the wing margin (WM; Figure  2, B and C). In general, protein kinases with a known function have a higher frequency of knockdown phenotypes than other kinases with less well-characterized functions (67% *vs* 40%, respectively). The phenotypes of gene knockdowns for kinases that have been previously characterized generally fits with the expectation. For example, knockdown of kinases regulating the phosphorylation and inactivation of Yorkie in the Hippo pathway result in wings larger than normal (Supplementary Figure S1B). Similarly, knockdown in components of the MAPK signaling pathway cause loss of veins and wing size-reduction phenotypes (Supplementary Figure S1C), whereas knockdown of genes belonging to the InR signaling pathway reduce the size of the wing without modifying the pattern of veins (Supplementary Figure S1E). Expected phenotypes were also observed for components of other signaling pathways such as Hedgehog (Supplementary Figure S1F), Notch (Supplementary Figure S1H), or Dpp (Supplementary Figure S1I), and for genes which activity is required for cell growth, division, adhesion, and survival (Supplementary Figure S1, D and J–I, respectively). These results suggest that the phenotypes of not previously characterized kinases in the wing disc would be informative as to their functional requirements.

**Table 1:** *Drosophila* kinases and phosphatases with a mutant wing phenotype after gene knockdown

Family	CG number	Name	H. Ortholog	Transformant	φ	f(x)	Ref.	GO
Kinases								
CHK	CG13369	CG13369	RBKS	100,747	EPL	—	—	MET
	CG3400	Pfrx	PFKFB3	25,959	S/V−	—	—	MET
IPK	CG45017	IP3K2	ITPKA-C	19,159	EPL/nec//S-P	Inositol hexakisphosphate substrate	Dean *et al.* (2015)	SIG
LK	CG10260	PI4KIIIα	PI4KA	105,614	S	SHW signaling	Yan *et al.* (2011)	CYT
	CG2929	Pi4KIIalpha	PI4K2A	110,687	V−(acv)	1-phosphatidylinositol 4 substrate	Burgess *et al.* (2012)	PTR
	CG31140	CG31140	DGKQ	101,347	WA(s)	—	—	MET
	CG3682	PIP5K59B	PIP5K1A	108,104	L	1-phosphatidylinositol-4-phosphate 5 substrate	Khuong *et al.* (2010)	SIG
	CG4141	Pi3K92E	PI3K92E	107,390	S(s)	Insulin signaling	Weinkove *et al.* (1999)	SIG
	CG6355	fab1	PIKFYVE	27,591	S(s)/WM	Secretory/endocytic pathway	Rusten *et al.* (2006)	PTR
	CG8657	Dgkepsilon	DGKE	4,659	S(s)	Diacylglycerol kinase activity	Frolov *et al.* (2001)	MET
	CG9985	sktl	PIP5K 57B6	101,624	L//S-P(s)	A/B cell polarity	Claret *et al.* (2014)	CYT
NUBCK	CG11811	CG11811	GUK1	110,740	LPL/S/WD	Guanylate kinase activity	Gaudet *et al.* (2011)	MET
	CG1725	dlg1	DLG1-4	109,274	S/WD	Polarity of larval imaginal cells	Bunker *et al.* (2015)	CA
	CG3140	Ak2	AK2	107,326	EPL/nec	Adenylate kinase activity	Gaudet *et al.* (2011)	MET
	CG32717	sdt	MPP5	100,685	WD	Zonula adherens assembly	Nam and Choi (2003)	CA
	CG5757	CG5757	DTYMK	110,460	nW//wt	Nucleoside diphosphate kinase activity	Gaudet *et al.* (2011)	MET
	CG5970	cbc	CLP1	100,686	LL/EPL	Polynucleotide 5'-hydroxyl-kinase activity	Gaudet *et al.* (2011)	RNA
	CG6364	Uck	UCK2	108,949	nW	Nucleoside kinase activity	FlyBase Curators (2004)	MET
	CG6612	Ak3	AK3	110,382	EPL/nec	Adenylate kinase activity	Gaudet *et al.* (2011)	MET
	CG9541	CG9541	AK5	102,912	WA	Cytidylate kinase activity	Gaudet *et al.* (2011)	MET
OK	CG10702	CG10702	INSRR	100,842	S(w)	Receptor tyrosine kinase activity	Gaudet *et al.* (2011)	CA
	CG12016	CG12016	NMRK1	103,613	S/WF(s)	Ribosylnicotinamide kinase activity	Gaudet *et al.* (2011)	MET
	CG1939	Dpck	DCAKD	100,276	EPL	Dephospho-CoA kinase activity	Gaudet *et al.* (2011)	MET
	CG3525	eas	ETNK1/2	103,784	S/WA/WM	Mushroom body development	Pascual *et al.* (2005)	MET
	CG5025	Sps2	SEPHS1-2	105,268	WF(s)/ds/S	Selenide, water dikinase activity	Gaudet *et al.* (2011)	MET
	CG8363	Papss	PAPSS1	110,544	EPL/nec/nW	Adenylylsulfate kinase activity	Gaudet *et al.* (2011)	MET
• Protein kinases								
AGC	CG10033	for	FOR/PKG	108,293	S/WA	Feeding behavior	Allen *et al.* (2017)	PRO
	CG10539	S6k	S6K	10539-R3	S(w)	Energy homeostasis	Allen *et al.* (2017)	SIG
	CG12069	CG12069	PRK	23,719	WA(s)	—	—	PRO
	CG12072	wts	WARTS	9,928	L//S(L)	SHW	Justice *et al.* (1995)	SIG
	CG1210	Pdk1	PDK1	18,736	S(s)/F	Insulin	Cho *et al.* (2001)	SIG
	CG17998	Gprk2	GPRK2	101,463	S-P(w)	Hh	Molnar *et al.* (2007)	SIG
	CG2049	Pkn	PKN1/3	108,870	V+/S/CD	Rho effector	Betson and Settleman (2007)	CYT
	CG4006	Akt1	AKT	103,703	S(s)	Insulin	Scanga *et al.* (2000)	SIG
	CG4012	gek	GEK	4012R2	S(L)	Actin	Luo *et al.* (1997)	CYT
	CG42783	aPKC	PRKCI/PRKCZ	105,624	nW//S-P(s)	A/B cell polarity	Kaplan *et al.* (2011)	CA
	CG4379	Pka-C1	PKA Cl	101,524	S-P	Hh/MAPK	Ohlmeyer and Kalderon (1998)	SIG
	CG6498	dop	MAST	35,100	WA(s)/V+	Tubulin	Hain *et al.* (2014)	CYT
	CG8637	trc	NDR	107923	S/V+/WA	Actin	Geng *et al.* (2000)	CYT
	CG9774	Rok	ROCK1	104,675	S/CD/WD	Actin	Mizuno *et al.* (1999)	CYT
A-PK	CG11859	RIOK2	RIOK2	109,296	LL/EPL/nec	Positive effect on glial cell proliferation	Read *et al.* (2013)	PRO
	CG17603	Taf1	TAF1	106,119	LL/EPL	Regulation of RNA polymerase II	Gaudet *et al.* (2011)	DNA
	CG3008	CG3008	103828	RIOK3	S-P	Maturation of SSU-rRNA	Gaudet *et al.* (2011)	RNA
	CG32743	nonC	SMG1	41,990	S	NMD pathway	Long *et al.* (2010)	RNA
	CG33554	Nipped-A	TRRAP	52,486	S-P(s)/CD	Histone acetylation	Gause *et al.* (2006)	DNA
	CG3608	Adck	ADCK1	BL42841	WF/WD	—	—	MET
	CG4252	mei-41	MEI41/FRP1	103,624	V+(w)	Cell cycle (DNA checkpoint)	Brodsky *et al.* (2000)	DNA
	CG5092	Tor	MTOR	5092-R2	S(s)	Insulin/TOR pathway	Hennig *et al.* (2006)	SIG
	CG5206	bon	TRIM24	101737	WM	chromatin organization	Beckstead *et al.* (2005)	PRO
	CG8808	Pdk	PDK	106,641	S(w)	glucose homeostasis	Gaudet *et al.* (2011)	MET
CAMK	CG10177	CG10177	—	107,848	S/WA/V+	Secretory/endocytic pathway	Zacharogianni *et al.* (2011)	PTR
	CG10895	lok	LOKI/CHK2	110,342	WA(s)/V+/S	DNA damage checkpoint	Xu *et al.* (2001)	DNA
	CG14305	CG14305	TSSK1B	107,848	S(w)	—	—	PRO
	CG1830	PhKgamma	PHKG1/2	110,638	WA(s)	—	—	PRO
	CG3051	AMPKalpha	SNF1A	106,200	WF/S(s)/WA/+	Metabolism	Lee *et al.* (2007)	SIG
	CG32666	Drak	DRAK1/2	107,263	nW//S-P(s)	Actin	Neubueser and Hipfner (2010)	CYT
	CG33519	Unc-89	SPEG	106,267	V(+)/WA	Muscle	Schnorrer *et al.* (2010)	CYT
	CG42347	sqa	MYLK1/2/3	101,640	V+(w)/WA	Actin	Tang *et al.* (2010)	CYT
	CG42856	Sik3	SIK1/2/3	39,864	V+/WA	Insulin	Choi *et al.* (2015)	MET
	CG4290	Sik2	SIK2	103,739	PL//wt	Energy homeostasis	Choi *et al.* (2011)	PRO
	CG43143	Nuak1	NUAK1	45,401	S/WM	Autophagy	Brooks *et al.* (2020)	MET
	CG4629	CG4629	NIM1K	26,574	WA	Glucose starvation	Gaudet *et al.* (2011)	MET
	CG5408	trbl	TRIB2	106,774	WA(s)	Insulin signaling	Das *et al.* (2014)	MET
	CG6703	CASK	CAKI	34,184	S/WA	NMJ	Sun *et al.* (2009)	PRO
	CG6715	KP78a	MARK1-3	26,722	S/WA(s)/V+	—	—	CYT
	CG7125	PKD	PRKD	106,255	L//S(s)/CD	Actin	Maier *et al.* (2006)	CYT
	CG8485	CG8485	SNRK	35,940	S	—	—	PRO
CKI	CG2028	CkIα	CKIα	110,768	L/nW//S(s)/WA(s)	Hh/Wnt/SWH	Lum *et al.* (2003)	SIG
	CG2577	CG2577	CSNK1A1	105,471	PL/S(s)/WA//S-P	—	—	PRO
	CG6386	ball	VRK1	108,630	S-P(s)/CD	Histone phosphorylation	Aihara *et al.* (2004)	DNA
	CG6963	gish	CKIγ	26,003	S/CD	Vesicle trafficking	Gaut *et al.* (2012)	PTR
	CG8878	CG8878	–	100,985	S-P(s)	EGFR/MAPK	Ashton-Beaucage *et al.* (2014)	SIG
CMGC	CG10498	Cdk2	CDK2/CDC2c	104,959	L/nW//S-P(s)/WA	Cell cycle	Chen *et al.* (2003)	DIV
	CG10572	Cdk8	CDK8	107,187	S/V+(w)	G1/S	Leclerc *et al.* (1996)	DIV
	CG11489	srpk79D	SRPK1-3	47,544	WA	NMJ	Jonhson *et al.* (2009)	PRO
	CG12559	rl	ERK1A	109,108	L//V−(s)/S(s)	Ras/MAPK	Brunner *et al.* (1994)	SIG
	CG17090	Hipk	HIPK1	108,254	S(s)/WM	Positive regulation of Wnt signaling	Lee *et al.* (2009)	SIG
	CG17520	CkIIα	CSNK2A1	BL31645	S-P(s)	Hh	Jia *et al.* (2010)	SIG
	CG2621	sgg	GSK3β	101,538	L//WA/Q+/V+	Wnt	Peifer *et al.* (1994)	SIG
	CG31003	gskt	GSK3β	25,641	S-P/WA	Male gamete generation	Kalamegham *et al.* (2007)	PRO
	CG3319	Cdk7	CDK7	103,413	S	Cell cycle	Larochelle *et al.* (1998)	DIV
	CG42273	mnb	MNB	28,628	S/V−	SHW/FoxO	Tejedor *et al.* (1995)	SIG
	CG42320	Doa	CLK2	19,066	L//S-P	Autophagy	Tang *et al.* (2018)	MET
	CG42366	CG42366	ICK/MAK	108,102	S(s)/WA(s)/V+	—	—	PRO
	CG4268	Pitslre	CDK11B	107,303	EPL/nec//wt	Positive regulation of Toll signaling	Kanoh *et al.* (2015)	SIG
	CG5072	Cdk4	CDK4/6	40,576	S/CD	JAK/STAT/TOR	Kim *et al.* (2017)	DIV
	CG5179	Cdk9	CDK9	103,561	L//S-P(s)/CD/WA	Histone methylation	Eissenberg *et al.* (2007)	DIV
	CG5363	Cdk1	CDK1/CDC2	106,130	L//S-P(s)/CD/WA	Cell cycle	Stern *et al.* (1993)	DIV
	CG7028	CG7028	PRP4	107,042	PL/nW//S-P(s)	Splicing	Herold *et al.* (2009)	RNA
	CG7393	p38b	MAPK14	108,099	WA(s) (29°)	MAPK cascade	Han *et al.* (1998)	IMM
	CG7597	Cdk12	CDK12/13	BL34838	S-P(s)	Transcription	Bartkowiak *et al.* (2010)	DNA
	CG7892	nmo	NEMO/NLK	104,885	V+(s)/WA(s)/S	Wg/Dpp	Zeng and Verheyen (2004)	SIG
O-PK	CG1098	Madm	NRBP1	101,758	S(s)	Cell growth and proliferation	Gluderer *et al.* (2010)	PTR
	CG1107	aux	GAK	16,182	L//S-P/WA	Clathrin	Hagedorn *et al.* (2006)	PTR
	CG11221	meng	SBK1	42,947	S/WF	Memory	Lee *et al.* (2018)	PRO
	CG1227	CG1227	MPSK/PSK	105,610	L//S-P	—	—	PRO
	CG12306	polo	POLO/PLK1	20,177	L/nW//S-P(s)/CD	Cell cycle	Carmena *et al.* (1998)	DIV
	CG14030	Bub1	BUB1	101,096	S/WA/WM	Cell cycle	Logarinho *et al.* (2004)	DIV
	CG2087	PEK	EIF2AK3	16,427	V+/WA(s)	—	—	PRO
	CG3068	aur	AURORA	108,446	S-P(s)	Cell cycle	Glover *et al.* (1995)	DIV
	CG32417	Myt1	MYT1	105,157	WA	Cell cycle	Price *et al.* (2002)	DIV
	CG32742	Cdc7	CDC7	40,715	S	Cell cycle	Stephenson *et al.* (2015)	DNA
	CG34412	tlk	TLK1	46,426	L//S(s)/V+	Cell cycle	Carrera *et al.* (2003)	DIV
	CG5790	CG5790	CDC7	45,044	S/V+	Cell cycle	Stephenson *et al.* (2015)	DNA
	CG6551	fu	FUSED	6551R3	S-P	Hh/Dpp	Robbins *et al.* (1997)	SIG
	CG6620	aurB	AURKA/B/C	104,051	S-P(s)/CD	Cell cycle	Giet *et al.* (2001)	DIV
	CG7177	Wnk	WNK1	106,928	S(s)	Wing disc development	Serysheva *et al.* (2013)	PRO
	CG7838	BubR1	BUB1	26,109	S/V+/CD	Cell cycle	Logarinho *et al.* (2004)	DIV
	CG9746	Vps15	PIK3R4	BL34092	V+	Autophagy	Lindmo *et al.* (2008)	SIG
STE	CG10295	Pak	PAK2	12,553	WA	AJ	Harden *et al.* (1996)	CYT
	CG11228	hpo	MST2	104,169	L//S(L)/WF	SHW	Udan *et al.* (2003)	SIG
	CG14217	Tao	TAO1	17,432	WF/V+//S(L, w)/V+	SHW	Poon *et al.* (2011)	SIG
	CG14895	Pak3	PAK3	107,260	S(L)	Cytoskeleton actin//MAPK	Mentzel and Raabe (2005)	CYT
	CG15793	Dsor1	SOR	40,026	nW//S(s)/V−	EGFR/MAPK	Tsuda *et al.* (1993)	SIG
	CG16973	msn	NIK	101,517	S/WA/V+(w)/CD	JNK	Su *et al.* (1998)	SIG
	CG18582	mbt	STE20	10,9880	S(w)/WA	AJ	Menzel *et al.* (2008)	CA
	CG4527	slik	SLK	43,784	S	Cell cycle	Hipfner and Cohen (2003)	DIV
	CG5169	GckIII	STLK3	49,558	S/V+(w)/CD	SJ	Song *et al.* (2013)	PRO
	CG7693	fray	STK39	106,919	S/WA	Ion homeostasis	Li *et al.* (2019)	PRO
	CG7717	Mekk1	MAP3K4	110,339	S	JNK	Inoue *et al.* (2001)	SIG
	CG9738	Mkk4	SEK1/MKK4	9738-R1	S	JNK	Han *et al.* (1998)	SIG
TKL	CG10776	wit	TGFBR2	42,244	WA(s)	BMP	Zheng *et al.* (2003)	SIG
	CG14026	tkv	BMPR1	862	S-P(s)	Dpp	Penton *et al.* (1994)	SIG
	CG1891	sax	ACVR1	1891-R3	V±/S	Dpp	Nellen *et al.* (1994)	SIG
	CG2272	slpr	MAP3K9/10	106,449	S/V+/WA	JNK	Stronach and Perrimon (2002)	SIG
	CG2845	phl	RAF	CG4803	S(s)/V−	EGFR/MAPK	Douziech *et al.* (2006)	SIG
	CG2899	ksr	KSR	110,621	S(s)/V−	EGFR/MAPK	Douziech *et al.* (2006)	SIG
	CG31421	Takl1	MAP3K7	BL55903	S	JNK	Wong *et al.* (2013)	SIG
	CG4803	Takl2	MAP3K7	104,701	V+/WA/S/N	—	—	PRO
	CG7904	put	TGFBR2	7904-R2	S-P(s)	Dpp	Ruberte *et al.* (1995)	SIG
	CG8224	babo	TGFBR1	106,092	WF(s)//S	TGFβ	Brummel *et al.* (1999)	SIG
	CG10079	Egfr	EGFR	10079-R2	nW//S(s)/V−	EGFR/MAPK	Livneh *et al.* (1985)	SIG
	CG14396	Ret	RET	843	WA(s)	Actin	Soba *et al.* (2015)	CA
	CG14992	Ack	TNK2	39,857	PL//S/V+(w)/WA	Negative regulation of hippo signaling	Schoenherr *et al.* (2012)	SIG
	CG18085	sev	SEV	107,048	S	MAPK	Baslet *et al.* (1991)	SIG
	CG18402	InR	INS RECEPTOR	992	S	Insulin	Yamaguchi *et al.* (1995)	SIG
	CG42317	Csk	CSK	32,877	WA(w)//S(L)	SRC/JNK/JAK-STAT	Read *et al.* (2004)	SIG
	CG44128	Src42A	SRC 42A	26,019	S(s)/V−	AJ	Shindo *et al.* (2008)	SIG
	CG7524	Src64B	SRC 64B	35,252	S(w)	Actin	Djagaeva *et al.* (2005)	SIG
	CG7525	Tie	—	7525-R2	S/V+/WF	Cell survival	Bilak *et al.* (2014)	SIG
	CG8222	Pvr	FLT1	105,353	PL/nW//S-P	EGFR/MAPK and TORC1	Tran *et al.* (2013)	SIG
Phosphatases								
5’N	CG4827	veil	NT5E	100,050	S(w)	5'-nucleotidase activity	Fenckova *et al.* (2011)	MET
AP	CG3292	Alp7	ALPPL2	19,989	PL/nec	—	—	MET
	CG5567	CG5567	PGP	106,981	WA	—	—	PRO
	CG8105	Alp11	ALPI	104,510	WA	—	—	CGh
LP	CG11437	CG11437	PPAP1-2	9,452	WA	—	—	MET
	CG11440	laza	PPAP2	42,592	S/V+/WA	Phototransduction	Garcia-Murillas *et al.* (2006)	MET
	CG8709	Lpin	LPIN3	107,707	WS(dp)/F	Lipid homeostasis		DNA
SP	CG3400	Pfrx	PFKFB	25,959	S/V−	—	—	MET
IPP	CG15743	CG15743	IMPAD1	42,686	S	—	—	SIG
	CG17029	CG17029	IMPA1/2	49,565	WA	Autophagy	Allen *et al.* (2020)	MET
	CG4123	Mipp1	MINPP1	101,634	S/V−(cv)/WD	Regulation of filopodium assembly	Cheng and Andrew, (2015)	MET
	CG42271	CG42271	INPP4A	100,176	WA (s)/V+	—	—	MET
	CG42283	5PtaseI	INPP5A	33,768	WA/V+/S	Autophagy	Allen *et al.* (2020)	MET
	CG5671	Pten	PTEN	35,731	S(L)	Insulin	Goberdhan *et al.* (1999)	SIG
	CG6562	Synj	SYNJ1/2	46,070	V+(w)	Synapsis	Dickman *et al.* (2005)	PTR
	CG9128	Sac1	SACM1L	44,376	LPL/nec	Cytoplasmic microtubule organization	Forrest *et al.* (2013)	SIG
	CG9389	CG9389	IMPA1/2	44,663	S(w)	Signaling	Gaudet *et al.* (2011)	SIG
	CG9784	CG9784	INPP5K/J	108,075	WA	Signaling	Gaudet *et al.* (2011)	SIG
HAD-NPP	CG1814	CG1814	NT5DC3	106,195	WA/WD	—	—	DNA
	CG3705	aay	PSPH	110,661	WD	—	—	MET
	CG5177	CG5177		103,024	LL/EPL/nec	NOT trehalose-phosphatase activity	Yoshida *et al.* (2016)	MET
	CG5567	CG5567	PGP	106,981	WA	—	—	PRO
• Protein phosphatases								
HAD-PP	CG12078	CG12078	CTDNEP1	101,274	WA	—	—	PRO
	CG12252	Fcp1	CTDP1	106,253	PL/nec	Polytene chromosome	Tombácz *et al.* (2009)	DNA
	CG1696	Dd	CTDNEP1	104,785	S(L)/V−(L4)	Imaginal disc wing vein specification	Liu *et al.* (2011)	PRO
	CG2713	ttm50	TIMM50	103,638	EPL/nec	Mitochondrion organization	Sugiyama *et al.* (2007)	TRA
C-PTP	CG14297	CG14297	ACP1	102,071	S/V−(w)/WA	—	—	PRO
	CG32697	Ptpmeg2	PTPN9	104,427	EPL/nec	Border follicle cell migration	Chen *et al.* (2012)	PRO
	CG33747	primo-2	ACP1	23,081	L/nW//S-P	—	—	PRO
	CG3954	csw	PTPN6, 11	108,352	V−/S/WA	EGFR/MAPK	Perkins *et al.* (1996)	SIG
	CG9181	Ptp61F	PTPN1-2	108,888	S(w)	EGFR/MAPK	Tchankouo *et al.* (2014)	PRO
	CG9311	mop	PTPN23	104,860	S/V+	MAPK/SHW	Gilbert *et al.* (2011)	SIG
DSP	CG10089	CG10089	DUSP15/22	17,991	S/V+	—	—	PRO
	CG13197	CG13197	DUSP11	105,122	S	—	—	PRO
	CG1395	stg	CDC25	17,760	L//S-P(s)	Cell cycle	Edgar and O’Farrell (1990)	DIV
	CG14080	Mkp3	DUSP7	23,911	V+(w)	EGFR/MAPK	Ruiz-Gómez *et al.* (2005)	SIG
	CG14211	MKP-4	DUSP12	104,884	L/nW//S-P	JNK	Sun *et al.* (2008)	SIG
	CG14411	CG14411	MTMR10	109,622	S(w)	NOT PTP activity	Hatzihristidis *et al.* (2015)	PRO
	CG1810	mRNA-cap	RNGTT	3,798	L//S-P(s)	Hh	Chen *et al.* (2017)	RNA
	CG3530	Mtmr6	MTMR6-8	26,217	S(w)	Cell cycle	Chen *et al.* (2007)	DIV
	CG3632	CG3632	MTMR4	110,167	WM(s)	Regulation of autophagy	Gaudet *et al.* (2011)	PRO
	CG4965	twe	CDC25A-C	46,064	V−	Meiosis	Alphey *et al.* (1992)	DIV
	CG7850	puc	DUSP10	3,018	L//S-P	JNK	Martín-Blanco *et al.* (1998)	SIG
PPM	CG17746	CG17746	PPM1A	100,178	WF(s)/S	—	—	PRO
	CG2984	Pp2C1	PPM1D	33,599	V/WM	—	—	PRO
PPP	CG10930	PpY-55A	PPP1CB	102,021	nW//wt	—	—	PRO
	CG12217	PpV	PPP6C	101,997	L//S-P	JNK	Chi *et al.* (2018)	PRO
	CG17291	Pp2A-29B	PPP2R1A	49,672	L//S-P(s)	Cell cycle	Goshima *et al.* (2007)	PRO
	CG2096	flw	PPP1CB	104,677	S/WM	Myosin	Kirchner *et al.* (2007)	PRO
	CG2890	PPP4R2r	PPP4R2	105,399	L//S/V+	Cell cycle	Chen *et al.* (2007)	PRO
	CG32505	Pp4-19C	PP4C	25,317	nW//S-P(s)	Cell cycle	Helps *et al.* (1998)	PRO
	CG5643	wdb	PP2A/wdb	101,406	S	Cell cycle	Chen *et al.* (2007)	PRO
	CG5650	Pp1-87B	PPP1CA-C	35,025	L//S-P	Cell cycle	Cohen (1997)	PRO
	CG6235	tws	PPP2R2A-D	34,340	S/V−/WA	Cell cycle	Brownlee *et al.* (2011)	PRO
	CG6593	Pp1α-96A	PPP1C A-C	27,673	nW//S-P(s)	Wg/Hh	Swarup *et al.* (2015)	PRO
	CG7109	mts	PPP2CA-B	35,171	nW//S-P	Wg/Hh/MAPK	Zhang *et al.* (2009)	PRO
	CG8402	PpD3	PPP5C	24,309	V+/WA	Cell cycle	Chen *et al.* (2007)	PRO
UN-PPP	CG14216	Ssu72	SSU72	104,388	WM(w)S(w)	Regulation RNA polymerase II	Werner-Allen *et al.* (2011)	RNA
R-PTP	CG10975	Ptp69D	PTPRC	27,090	S/CD	Axon guidance	Desai *et al.* (1997)	PRO
	CG6899	Ptp4E	PTPRB	1,012	S	Axon guidance	Jeon *et al.* (2008)	PRO

Protein family (Family), CG number, gene name (Name), human ortholog (H. Ortholog), transformant RNAi line (Transformant), wing knockdown phenotype (Φ) and main described function [f(x)], representative reference (Ref.), and molecular classification (GO) into the classes general sugar and lipid metabolism (MET), signaling (SIG), cytoskeleton organization (CYT), protein transport across membranes (PTR), cell adhesion (CA), RNA biology (RNA), DNA biology (DNA), protein modifications (PRO), cell division (DIV), immunological responses (IMM), and solute transport (TRA). The abbreviations used to describe each phenotype are described in the main text. The list of references is presented as Supplementary material.

We were able to identify a phenotype for 40% of protein kinases not previously characterized in the *Drosophila* wing. These phenotypes could now be used as an entry point to perform a more detailed functional characterization of the corresponding genes and proteins. Despite the high fraction of genes that knockdown results in wings with altered morphogenesis, there are still many cases of genes expressed in the wing disc and for which we could not detect a mutant phenotype upon expression of the corresponding RNAi (208 genes). The reason for this result could be either a genuine lack of requirement of the gene during wing development, gene redundancy in those cases where multiple kinases affect a similar set of targets, or insufficient reduction in the level of mRNA following the RNAi knockdown. Focusing on those cases in which the expression of RNAi results in wings with altered size and/or vein patterns, we did not find a particular phenotypic enrichment for a given family of protein kinases (Figure  2C). Many of the phenotypes we found are reminiscent of those caused by alterations of specific signaling pathways in the wing. For example, knockdown of *genghis khan* (*gek*), the fly orthologous to human CDC42 binding protein kinase alpha, results in wings larger than normal (Figure  2F), similar to increased Yorki activity. The Gek protein is a putative effector for *Drosophil*a Cdc42, which promotes Actin polymerization during *Drosophila* oogenesis (Luo *et al.* 1997), and the Actin cytoskeleton is a key mediator of the regulation of Hippo signaling (Seo and Kim 2018). In contrast, loss of Ret reduces wing size and causes a wing blisters (Figure  2E), which is compatible with the requirement of the gene in extracellular matrix adhesion during dendrite development (Soba *et al.* 2015). Loss of *cdk12*, encoding a transcription elongation-associated CTD kinase (Bartkowiak *et al.* 2010), results in ectopic vein formation and loss of wing margin structures reminiscent of loss of Notch signaling (Figure  2G). Strong effects in wing size and pattern were observed upon knockdown of several kinases such as Cdk9 (Supplementary Figure S2), which is involved in RNA polymerase II elongation control (Peng *et al.* 1998), CKIalpha (Fig.  2H), which is involved in multiple signaling pathways (see, *e.g.*, Apionishev *et al.* 2005) and nonC (Supplementary Figure S3), related to the nonsense-mediated mRNA decay pathway (Rehwinkel *et al.* 2005). Other protein kinases affecting the veins may do so by altering the early secretory pathway (CG10177 in Supplementary Figure S4, see Zacharogianni *et al.* 2011), the endocytic pathway (Vps15; Supplementary Figure S3; see O’Farrell *et al.* 2017), or gene expression, such as Cdk8 (Supplementary Figure S2; see Loncle *et al.* 2007) and CG8878 (Fig.  2I; see McCracken and Locke 2014). Knockdown of other kinases with totally unknown function such as Nuak1 (S/WM: Supplementary Figure S4), CG1227 (S-P; Supplementary Figure S3), RIOK1 (S/WA; Supplementary Figure S3), and CG2577 (S-P; Supplementary Figure S3) also affect wing development in specific ways. The full collection of wings showing a phenotype distinct to wild type is shown in Supplementary Figures S1–S5.

### Phenotypic screen of phosphatases in the wing

**Figure 3:**
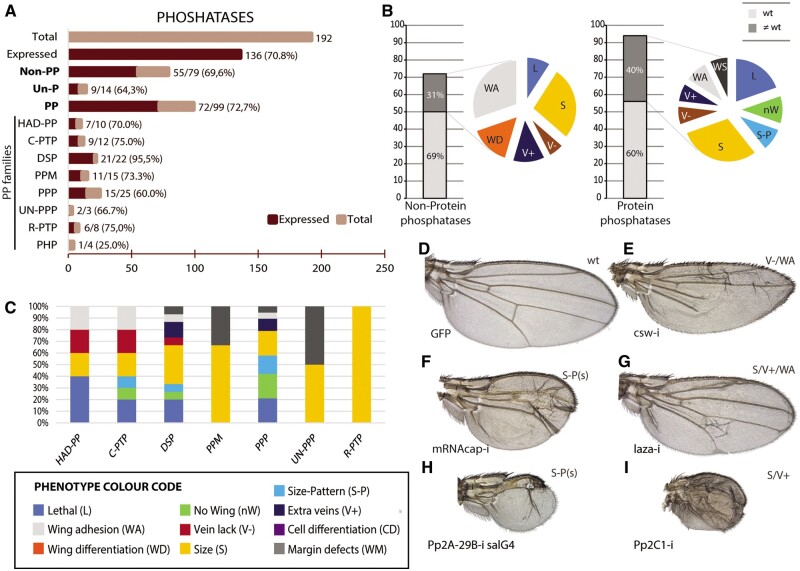
Global results of the RNAi screen for the complement of *Drosophila* phosphatases. (A) Fraction of phosphatase genes expressed in the wing disc separated into the groups nonprotein phosphatases (Non-PP; 79 genes), unclassified phosphatases (Un-P; 14 genes), and protein phosphatases (PP; 99 genes). Protein phosphatases were further subdivided into the groups serine-threonine protein phosphatases of the classes HAD, PPP, PPM, and unclassified (HAD-PP, PPP, PPM, and Un-PPP, respectively), Tyrosine phosphatases, including cytosolic (C-PTP) and receptor proteins (R-PTP), Histidine phosphatases (PHP), and DSP. (B) Number of nonprotein phosphatases (left) and protein phosphatases (right) for which we tested its knockdown phenotype, and fraction of genes with a mutant phenotype (dark gray section) or without any phenotype (light gray section) in knockdown conditions. Colored sectors show the percentage of each phenotype for nonprotein phosphatases (left) and for protein phosphatases (right). Lethality (L; dark blue sector), loss of wing (nW; green sector), changes in wing size and pattern (S-P; light blue sector), changes in size (S; yellow sector), loss of veins (V−; red sector), extra or thicker veins (V+; dark blue sector), wing adhesion defects (WA; gray sector), trichome differentiation or size defects (CD; purple sector), and other phenotypes (WS; dark gray sector). (C) Percentage of lethal (blue) and visible mutant phenotypes respect the total number of observed phenotypes in the HAD-PP, C-PTP, DSP, PPM, PPP, UN-PPP, and R-PTP classes. (D) Control *nub-Gal4/UAS-GFP* wing. (E–I) Representative mutant wings (E) *UAS-Dicer2; nub-Gal4/UAS-csw*-*RNAi* wing (csw-i) showing the expected loss of veins phenotype. (F) *UAS-Dicer2/+; sal^EPv^-Gal4/UAS-mRNAcap-RNAi* (mRNAcap-i)*.* (G) *UAS-Dicer2/+; nub-Gal4/UAS-laza-RNAi* (laza-i). (H) *UAS-Dicer2/+; sal^EPv^-Gal4/UAS-Pp2A28B-RNAi* (Pp2A-28B-i). (I) *UAS-Dicer2/+; nub-Gal4/UAS-Pp2C1-RNAi* (Pp2C1-i).

Phosphatases catalyze the hydrolysis of a phosphate group from a given substrate. We included in our analysis 79 nonprotein phosphatases, 99 protein phosphatases, and 14 unclassified phosphatases (Figures  1 and 3A). These genes are expressed in the wing disc with percentages varying from 64% for unclassified phosphatases to 73% for protein phosphatases (Figure  3A). Nonprotein phosphatases include proteins with broad substrate specificity (acid and alkaline phosphatases), lipid phosphate phosphatases (LPP), which are integral membrane proteins that catalyze the dephosphorylation of a variety of lipid phosphates, phosphatidylinositol lipid phosphatases, sugar phosphatases, and HAD family nonprotein phosphatases. The genes CG9115, CG3632, CG3530, and CG5026, which have Phosphoinositide 3 phosphatase activity, also have Dual-Specificity Phosphatases (DSP) activity, and they were classified in this last group. A large fraction of these genes (88%) is related to metabolism (Supplementary Table S1). The frequency of lethality or wing mutant phenotype for this group of genes is low (31%; Figures  1 and 3B), and is only above average for phosphatidylinositol lipid phosphatase enzymes (45%; Table  1). These proteins remove phosphate groups from positions 3, 4, or 5 of inositol molecules, participating in the metabolism of phosphoinositides. Although these lipids bind a variety of target proteins mediating cell membrane functions including vesicular trafficking, signaling, and cytoskeletal function (Balakrishnan *et al.* 2015) phosphatidylinositol lipid phosphatases were classified mostly in the metabolism class.

Protein phosphatases (99 members) belong to four groups: Haloacid Dehalogenases (HAD-PP; Burroughs *et al.* 2006), Histidine phosphatases and the more numerous Serine/Threonine Phosphatases and Tyrosine phosphatases (Morrison *et al.* 2000; Hatzihristidis *et al.* 2015). These genes are generally expressed in the wing disc (73%, Figure  3A), ranging from 60% in the case of Serine/Threonine Phosphatases of the PPP group to 96% for DSP (Figures  1 and 3A). Some DSP can also dephosphorylate nonprotein targets including phosphoinositide, RNA 5'-triphosphate, and carbohydrates (Hatzihristidis *et al.* 2015).

The frequency of *nub-Gal4/UAS-RNAi* combinations with a lethal or altered wing phenotype for protein phosphatase genes was 40% (Figure  3B), reaching higher values for cytoplasmic tyrosine phosphatases (60%; Figure  1) and DSP (52%; Figure  1). For proteins with a known function the phenotype was as expected. For example, *csw*, acting downstream of receptor tyrosine kinases (Johnson Hamlet and Perkins 2001), displayed a loss of vein phenotype (Figure  3E), and *mRNA-CAP*, which regulates Hh signaling through antagonizing PKA (Chen *et al.* 2017) has strong size and pattern effects (Figure  3F). Inositol and Lipid phosphatases, such as *5PtaseI and laza* (Figure  3G), display a similar extra-vein phenotype, suggestive of increased EGFR signaling. Both of them also have adhesion defects between dorsal and ventral surfaces of the wing (WA phenotype). This is a common feature of the knockdown of other phosphoinositide phosphate phosphatases such as CG9784, CG11477, and CG17029 (Supplementary Figure S6). Particularly strong phenotypes were observed in the case of genes encoding different subunits of the protein phosphatase type 2A complex (PP2A), which modulates the insulin (Kulkarni *et al.* 2016), Hedgehog (Su *et al.* 2011), and Wingless (Luo *et al.* 2007) signaling pathways. For example, knockdown of *Pp2A-29B*, encoding the structural A subunit of PP2A phosphatase enzyme (Chen *et al.* 2007) prevents wing development (Figure  3H). A similar phenotype is observed in *Pp1α-96A* knockdown flies (Supplementary Figure S6). This protein also has multiple functions including the regulation of the Hedgehog and Wingless signaling pathways (Su *et al.* 2011). The knockdown of several PPP Serine/Threonine phosphatases results in lethality (*nub-Gal4*) and defects in wing size and pattern (*sal^EPv^-Gal4*) with a phenotype similar to *Pp2A-29B* knockdown (Figure  3H). Some examples are *mts*, *Pp1-87B*, *Pp1alpha-96A*, *Pp4-19C*, *PPP4R2r*, a component of the protein phosphatase 4 complex that may coordinate centrosome maturation and cell migration (Chen *et al.* 2007), *Pp2A-29B and PpV*, encoding the catalytic subunit of PP6 [Supplementary Figure S6, PPP family and see Ma *et al.* (2017)]. A similar strong phenotype, in which all the central domain is differentiated as vein tissue, is also observed for *Pp2C1* (Figure  3I). In contrast, knockdown of the protein tyrosine phosphatases *Ptp69D and Ptp4E*, which might mediate negative regulation of the receptors EGFR, Breathless, and Pvr (Jeon *et al.* 2012), results only in defects in wing size (Supplementary Figure S7). The DUSP family offers a wide range of wing phenotypes including extra veins (*CG10089*), lack of veins (*twe*), size defects (*CG13197*, *Mtmr6*), and severe size and pattern defects (*stg*, *mRNAcap*, *Mkp4 and Puc*). The complete collection of phenotypes for protein and inositide phosphatases is shown in Supplementary Figure S6 and S7.

### Developmental bases for “wing size” and “wing size and pattern” defects

**Figure 4:**
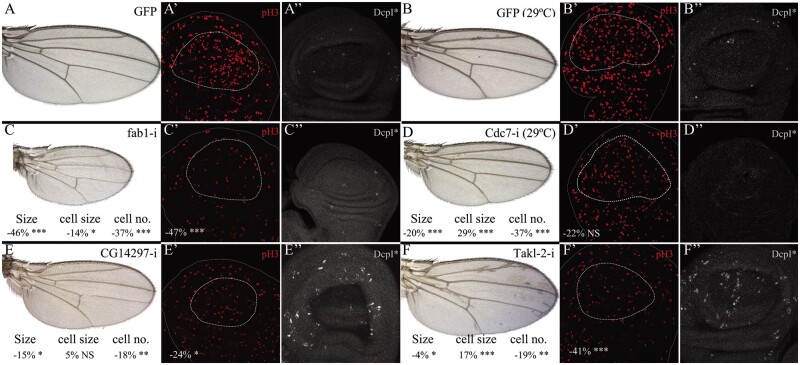
Cell proliferation and viability of genetic combinations affecting wing size. (A-B) Wing phenotype (A and B), expression of phospho-Histone3 (pH3; red in A’ and B’) and cleaved-Dcp1 (DcpI*, white in A’’ and B’’) in control *UAS-Dicer2; nub-Gal4/UAS-GFP* third instar wing discs grown at 25°C (A–A’’) and 29°C (B–B’’). (C–F) Wing phenotype (C–F), expression of phospho-Histone3 (pH3; red in C’–F’), and cleaved-Dcp1 (DcpI*, white in C’’–F’’) in the genetic combinations *UAS-Dicer2; nub-Gal4/UAS-fab1-RNAi* (C–C’’), *UAS-Dicer2; nub-Gal4/UAS-cdc7-RNAi* (D–D’’), *UAS-Dicer2; nub-Gal4/UAS-CG14297-RNAi* (E–E’’), and *UAS-Dicer2; nub-Gal4/UAS-Takl2-RNAi* (F–F’’). Below each wing is indicated the percentage of wing size (Size), cell size (cell size), and wing cell number (cell no.) modification for each genetic combination compared to their control *UAS-Dicer2; nub-Gal4/UAS-GFP* wings.

The most common phenotypes observed in *UAS-RNAi/nub-Gal4 and UAS-RNAi/sal^EPv^-Gal4* combinations are those in which the size of the wing is altered, most frequently reduced (see, *e.g.*, Figures  2E and 3, E, G, and I). This phenotype could be caused by a reduction in the number of wing cells (due to cell death or reduced cell division in the imaginal disc), by a reduction in the size of the cells, or by a combination of these two effects. We analyzed cell division (mitotic index) and death in the wing imaginal disc and cell size in the adult wing for four genetic combinations with different degrees of wing size reduction (Figure  4). In wild-type imaginal discs, cell division (mitosis) occurs throughout the presumptive wing blade and cell death is only testimonial and scattered in the disc (Figure  4, A and B). In the combinations analyzed the mitotic index in the wing pouch region was reduced, from 47% (*nub-Gal4/UAS-fab1-RNAi*; Figure  4C) to 24% (*nub-Gal4/UAS-CG14297-RNAi*; Figure  4E). Cell size in the adult wing was also generally reduced, from 29% (*nub-Gal4/UAS-Cdc7-RNAi*; Figure  4D) to 14% (*nub-Gal4/UAS-fab1-RNAi*; Figure  4C). The occurrence of cell death in wing discs corresponding to smaller adult wings was generally low (Figure  4, C–F). These observations suggest that reduced wing size is mostly due to a lower rate of mitosis accompanied by different degrees of cell size reduction.

**Figure 5:**
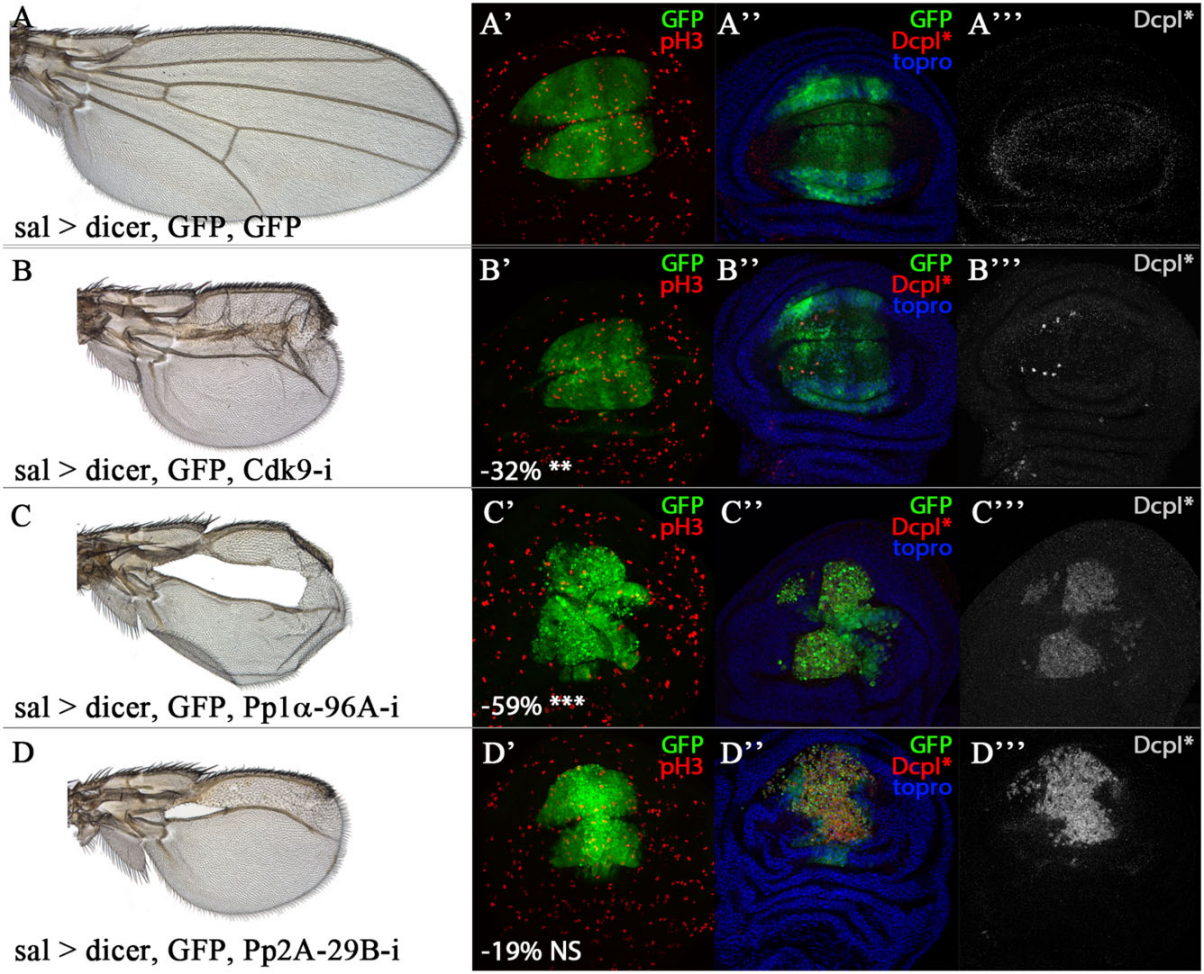
Cell proliferation and viability of genetic combinations affecting wing size and pattern. (A) *UAS-Dicer2; sal^EPv^-Gal4 UAS-GFP/UAS-GFP* control wing. (A’–A’’’) Late third instar wing disc of *UAS-Dicer2; sal^EPv^-Gal4 UAS-GFP/UAS-GFP* genotype showing the expression of GFP (GFP; green in A’–A’’), phospho-Histone 3 (pH3; red in A’), cleaved-Dcp1 (white in A’’’), and Topro3 (topro; blue in A’’). (B) Adult female wings of *UAS-Dicer2; sal^EPv^-Gal4 UAS-GFP/UAS-Cdk9-RNAi*. (B’–B’’’) Late third instar wing disc of *UAS-Dicer2; sal^EPv^-Gal4/UAS-Cdk9-RNAi* genotype showing the expression of GFP (green in B’–B’’), phospho-Histone 3 (pH3; red in B’), cleaved-Dcp1 (DcpI*; white in B’’’), and Topro3 (topro; blue in B’’). (C) *UAS-Dicer2; sal^EPv^-Gal4 UAS-GFP/UAS-Pp1α96A-RNAi*. (C’–C’’’) Late third instar wing disc of *UAS-Dicer2; sal^EPv^-Gal4/UAS-Pp1α96A-RNAi* genotype showing the expression of GFP (GFP; green in C’–C’’), phospho-Histone 3 (pH3; red in C’), cleaved-Dcp1 (DcpI*; white in C’’’), and Topro3 (topro; blue in C’’). (D) *UAS-Dicer2; sal^EPv^-Gal4 UAS-GFP/UAS-Pp2A29B-RNAi*. (D’–D’’’) Late third instar wing disc of *UAS-Dicer2; sal^EPv^-Gal4 UAS-GFP/UAS-Pp2A29B-RNAi* genotype showing the expression of GFP (green in D’–D’’), phospho-Histone 3 (pH3; red in D’), cleaved-Dcp1 (DcpI*; white in D’’’), and Topro3 (blue in H’). Below the wing discs shown in B’, C’, D’ percentage of mitotic index reduction for each genetic combination compared to their control *UAS-Dicer2; sal^EPv^-Gal4 UAS-GFP/UAS-GFP* discs.

The second most frequent class of mutant phenotypes includes strong changes in the size of the wing accompanied by alterations in the pattern of veins. For many of these cases, the expression of RNAi in the entire wing (*nub-Gal4*) resulted in PL, and the effects in the wing could only be analyzed in combinations with the weaker driver *sal^EPv^-Gal4* (Table  1). We analyzed cell death and mitosis in three *sal^EPv^-Gal4/UAS-RNAi* combinations leading to the formation of small wings with aberrant venation patterns and found that some but not all of them are accompanied by massive cell death in the wing disc (Figure  5). This result indicates that the corresponding genes are required for cell viability and suggest that many genetic combinations in which the size and pattern of the wing are severely affected are a consequence of continuous and massive cell death in the imaginal disc epithelium.

### Quantitative changes in the activity of the EGFR signaling pathway are translated into phenotypic series affecting wing vein formation and wing size

**Figure 6:**
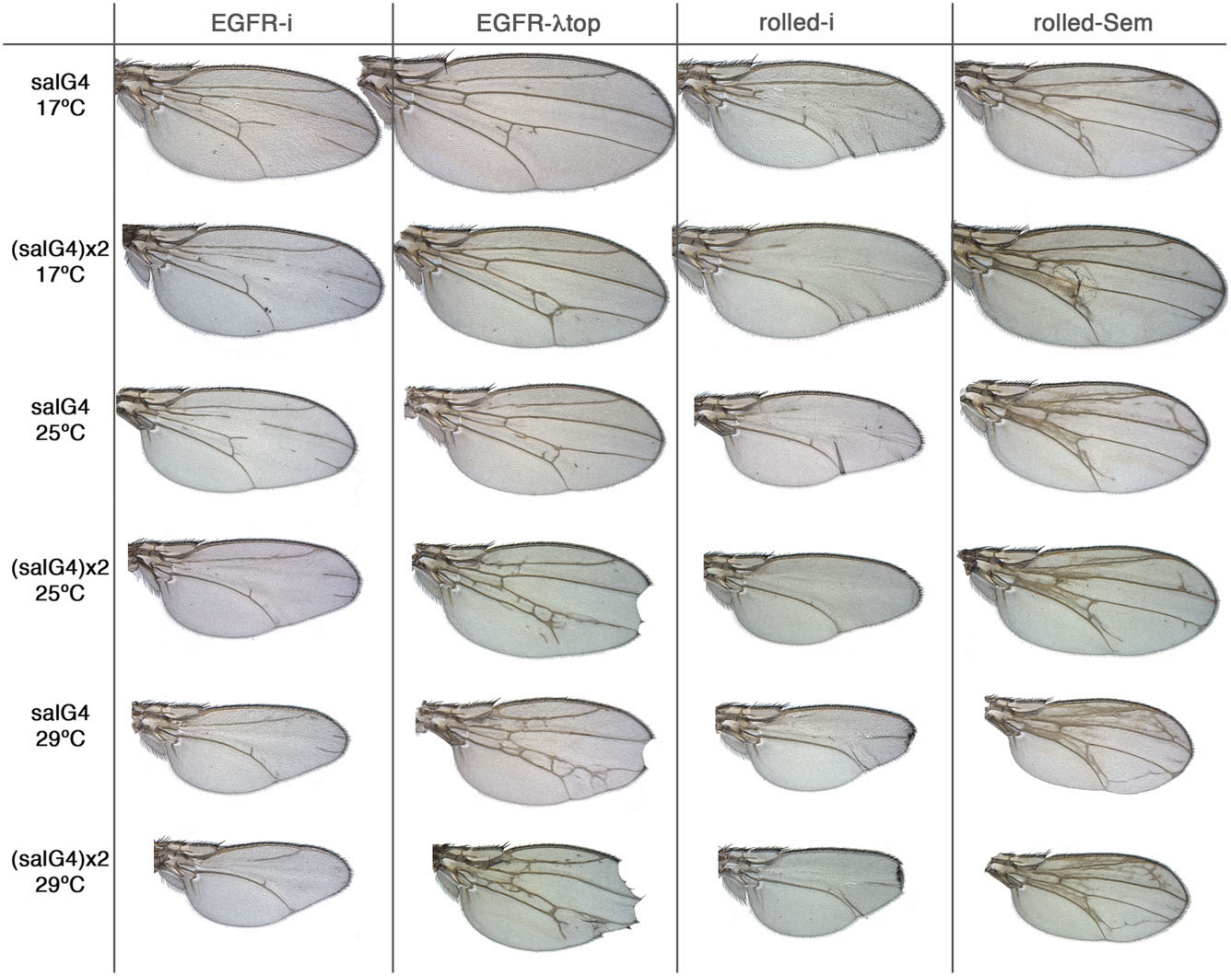
Phenotypic series of increased and reduced EGFR signaling in the adult wing. Wings from females grown at 17°C, 25°C, and 29°C (indicated in the left column) of genotypes containing one (salG4) or two [(salG4)x2]) copies of the *sal^EPv^-Gal4* driver in combination with *UAS-EGFR-RNAi* (EGFR-i column), *UAS-EGFR^λtop^* (EGFR-λtop column), *UAS-rl-RNAi* (rolled-i column), and *UAS-rl^Sem^* (rolled-Sem column). Note how the severity of each mutant wing increases (top to bottom) with the level of Gal4/UAS expression.

The EGFR signaling pathway contributes to the regulation of imaginal cell division, growth, viability, and differentiation (Shilo 2003). The pathway includes a Tyrosine kinase transmembrane protein as receptor (EGFR) and several protein kinases and phosphatases that participate as core components of the receptor intracellular signal transduction cascade (Shilo 2003). In order to search for additional protein kinases and phosphatases that could impinge on the EGFR signaling cascade, we used genotypes in which the activity of the pathway is modified at the level of the receptor or at the level of the MAP kinase ERK (*rolled*). For both EGFR and ERK, we aimed to modify the phenotype resulting from higher than normal activation (*EGFR^λtop^ and rolled^sem^*, respectively) or by lower than normal activation (*EGFR-RNAi and rolled-RNAi*, respectively) by the coexpression of RNAi’s targeting all protein kinases and phosphatases. As a preliminary experiment, we generated genotypes with different degrees of EGFR and ERK variants overexpression. To do this, we changed the number of doses of the Gal4 insertions used and also the temperature at which the flies were raised. We were able to establish for each case a clear phenotypic series of effects, suggesting a linear translation between EGFR signaling output and wing phenotype (Figure  6). For example, in the cases of EGFR pathway insufficiency caused by the expression of RNAi directed against EGFR or ERK the wing becomes progressively smaller as the level of RNAi expression increases (Figure  6A, *EGFR-i and rolled-i* columns). Simultaneously, the number of veins is also progressively reduced, from small gaps in the L4 vein (low expression of RNAi, upper panels in Figure  6) to the absence of all the veins included in the domain of *sal^EPv^-Gal4* expression (L2, L3, and L4; high expression of RNAi; lower panels in Figure  6A). Conversely, expression of activated forms of EGFR (EGFR-λtop) or ERK (Rolled^Sem^) results in the differentiation of ectopic veins and wing size reduction, and these phenotypes are stronger in genotypes with maximal overexpression (Figure  6, second and fourth columns). We expect that changes on the level of EGFR or ERK activity, caused by knockdown of other genes, will modify the background phenotype of each individual combination along similar phenotypic series.

### Modifier screen of kinases and phosphatases in EGFR mutant backgrounds

**Figure 7:**
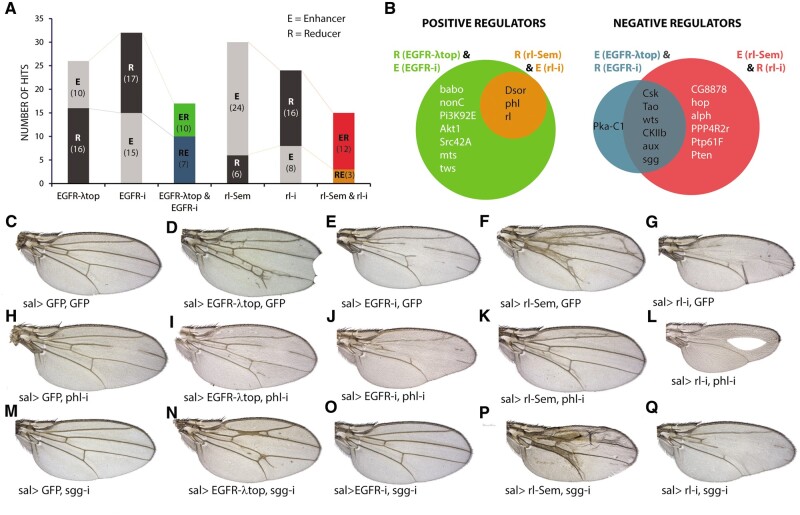
Modifications of EGFR and ERK phenotypes by knockdown of kinases and phosphatases. (A) Number of genes that behave as enhancers (E; gray section) or suppressors (R; black section) in the following genetic combination: *sal^EPv^-Gal4/UAS-RNAi; UAS-EGFR^λtop^/+* (EGFR-λtop), *sal^EPv^-Gal4 UAS-EGFR-RNAi/UAS-RNAi* (EGFR-i), *sal^EPv^Gal4 UAS-rl^Sem^/UAS-RNAi* (rl-Sem), and *sal^EPv^-Gal4 UAS-rl-RNAi/UAS-RNAi* (rl-i). Colored columns represent the number of genes identified as EGFR-λtop enhancers and EGFR-i suppressors (ER; green), EGFR-λtop suppressors and EGFR-i enhancers (RE; blue), rl-Sem enhancers and rl-i suppressors (ER; red) and rl-Sem suppressors and rl-i enhancers (RE; orange). In brackets the number of genes in each class. (B) Genes identified simultaneously in both EGFR and rl screens as positive regulators (green and orange circles, respectively) and as negative regulators (blue and red circles, respectively). (C–G) Control phenotypes used as a background to screen for modifiers UAS-RNAi lines. (H–L) Example of *phl*, a known member of the EGFR signaling pathway, in the combinations *sal^EPv^-Gal4 UAS-GFP/UAS-phl-RNAi* (H), *sal^EPv^-Gal4 UAS-EGFR^λtop^/UAS-UAS-phl-RNAi* (I), *sal^EPv^-Gal4 UAS-EGFR-RNAi/UAS-phl-RNAi* (J), *sal^EPv^-Gal4 UAS-rl^Sem^/UAS-phl-RNAi* (K) and *sal^EPv^ UAS-rl-RNAi/UAS-phl-RNAi* (L). (M-Q) Adult wings of combinations involving *UAS-sgg-RNAi*: *sal^EPv^-Gal4 UAS-GFP/UAS-sgg-RNAi* (M), *sal^EPv^-Gal4 UAS-EGFR^λtop^/UAS-UAS-sgg-RNAi* (N), *sal^EPv^-Gal4 UAS-EGFR-RNAi/UAS-sgg-RNAi* (O), *sal^EPv^-Gal4 UAS-rl^Sem^/UAS-sgg-RNAi* (P), and *sal^EPv^ UAS-rl-RNAi/UAS-sgg-RNAi* (Q).

We crossed a collection of UAS-RNAi targeting protein and inositide kinases (211 genes; Supplementary Table S2) and phosphatases (88 genes; Supplementary Table S2) into four different genetic backgrounds with higher (*UAS-EGFR-λTop/+; sal^EPv^Gal4/+ and sal^EPv^-Gal47+; UAS-rl^sem^*/+; Figure  7) or lower (*sal^EPv^-Gal4/+; UAS-EGFR-RNAi/+ and sal^EPv^-Gal4 UAS-rl-*RNAi/+ Figure  7) than normal EGFR signaling pathway activity. From the resulting phenotypes, we identified those which consistently increased the background wing size and vein differentiation phenotypes (enhancers) and those which reduced these phenotypes (suppressors). In most cases, the expression of UAS-RNAi lines resulted in additive phenotypes (89% for kinases and 91% for phosphatases in average; see Supplementary Table S2). We found modifiers in cases of genes which knockdown have a phenotype by itself (26 genes; Supplementary Table S2) and also for genes which knockdown does not affect wing development (22 genes). In general, the modifiers affected one (11 genes) or more than one background phenotype (24 genes), with cases in which two (6 genes), three (9 cases), or the four (9 cases) backgrounds we used were modified by the knockdown (Supplementary Table S2). Consistently, genes acting as enhancers of EGFR gain of activity conditions usually behave as suppressors of EGFR knockdown conditions and *vice versa* (Figure  7, A and B). Not unexpectedly, the genes with more hits correspond to core members of the EGFR signaling pathway (*Dsor*, *phl*, and *rl*; Figure  7, B and H–L). Other genes identified as positive regulators because of the opposite effects of their knockdown on the EGFR-λTop and EGFR-RNAi phenotypes, are members of other signaling pathways (*babo*, *Akt1*, *PI3K92E*, and *mts*), phosphatidylinositol 3-kinases (*nonC*), cytoplasmic tyrosine kinases (*Src42A*), and a regulatory subunit of the protein phosphatase 2A (*tws*; Figure  7B). Similarly, genes identified as negative regulators of EGFR signaling are either components of other signaling pathways (*hop*, *Ptn*, *csk*, *wts*, *Tao*, *alph*, and *sgg*; Figure  7, B and M–K for the case of *sgg*), and also include a regulator of clathrin dynamics (*aux*; Hagedorn *et al.* 2006), Casein kinase II β subunit (an enhancer of position effect variegation, see McCracken and Locke 2014) and the phosphatases *protein phosphatase 4 regulatory subunit 2-related* (*PPP4R2r*) and *Ptp61F* (Figure  7B).

### The components of the InR pathway modify consistently the phenotypes of loss and gain of InR activity

**Figure 8:**
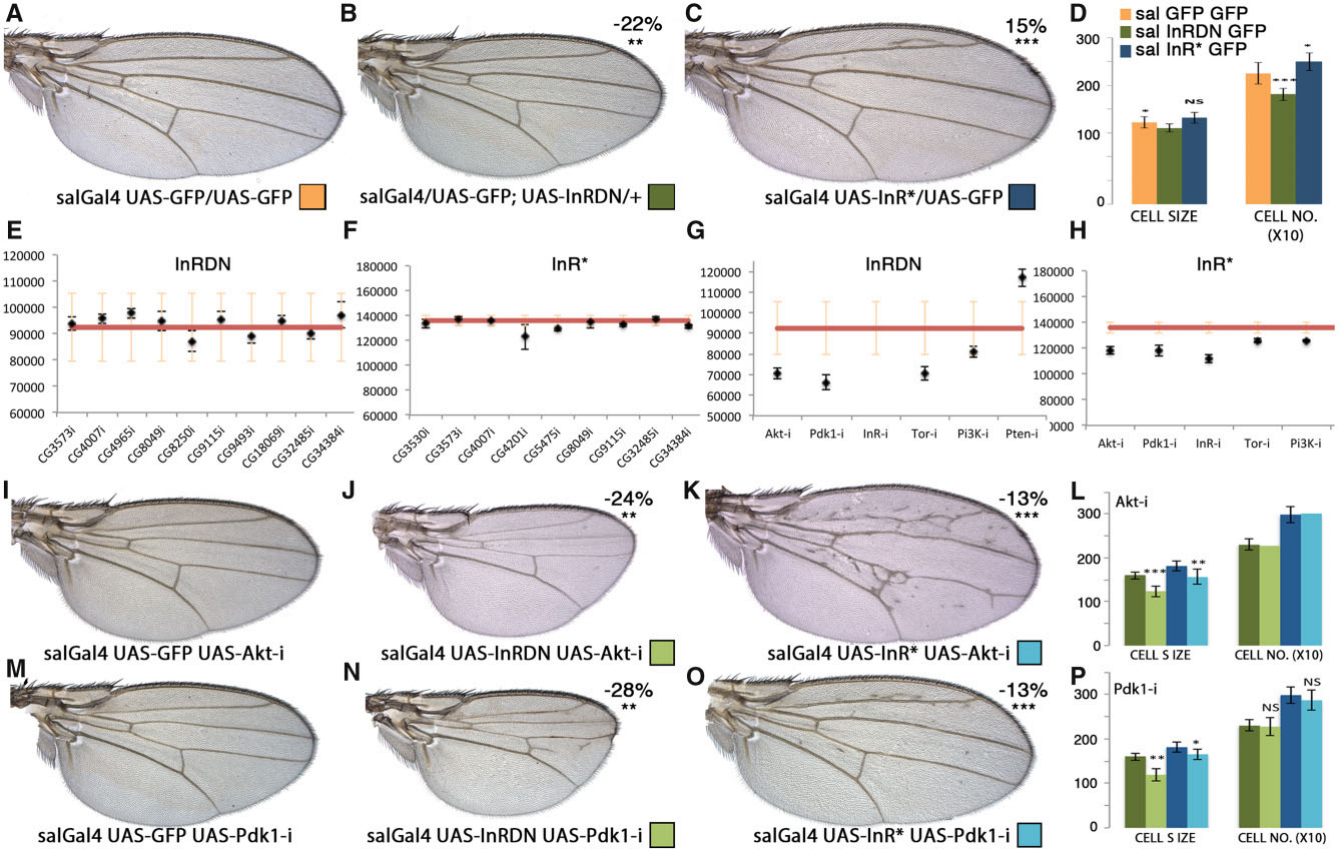
Wing phenotypes resulting from altered levels of InR signaling pathway components. (A–C) Control *sal^EPv^-Gal4 UAS-GFP/UAS-GFP* wing (A; orange code) and wings of *sal^EPv^-Gal4 UAS-GFP/UAS-InR^DN^* (B; green code), and *sal^EPv^-Gal4 UAS-GFP/UAS-InR^Act^* (C; blue code). The change in wing size of combinations involving *InR^DN^ and InR^Act^* relative to control wings is indicated in the upper-right corner. (D) Quantification of cell size (CELL S) and cell number (CELL NO.) of wings illustrated in (A–C). (E, F) Wing size of ten *sal^EPv^-Gal4 UAS-InR^DN^/UAS-RNAi* (InRDN; E) and nine *sal^EPv^-Gal4 UAS-InR^Act^/UAS-RNAi* (InR*; F) combinations that were selected random among those without effects on the *InR^DN^* or *InR^Act^* genetic backgrounds. (G, H) Wing size of six *sal^EPv^-Gal4 UAS-InR^DN^/UAS-RNAi* (G) and five *sal^EPv^-Gal4 UAS-InR^Act^/UAS-RNAi* (H) combinations involving known members of the InR pathway (*UAS-Akt-RNAi*, *UAS-PKB-RNAi*, *UAN-InR-RNAi*, *UAS-PI3K-RNAi*, and *UAS-Pten-RNAi*). (I) Adult wings of genetic combinations involving UAS-Akt-RNAi combinations: *sal^EPv^-Gal4 UAS-GFP/UAS-Akt-RNAi* (left), *sal^EPv^-Gal4 UAS-InR^DN^/UAS-Akt-RNAi* (middle), and *sal^EPv^-Gal4 UAS-InR^Act^/UAS-Akt-RNAi* (right). (J) UAS-Pdk1 combinations: *sal^EPv^-Gal4 UAS-GFP/UAS-Pdk-RNAi* (left), *sal^EPv^-Gal4 UAS-InR^DN^/UAS-Pdk-RNAi* (middle), and *sal^EPv^-Gal4 UAS-InR^Act^/UAS-Pdk-RNAi* (right). The wing cell size (CELL S) and number (CELL NO.) are shown to the right with the columns in the same color code as the pictures shown in (I) and (J).

InR signaling is required for wing imaginal cells growth and cell division (Edgar 2006). Consistently, expression of dominant negative or constitutively activated forms of the InR in the wing disc (*sal^EPv^-Gal4/UAS-GFP; UAS-InR^DN^/+ and sal^EPv^-Gal4 UAS-InR^Act^/UAS-GFP*) results in the formation of smaller and larger wings, respectively (Figure  8, A–C). These wings are formed by less and smaller cells (InR^DN^) or by more and larger cells (InR*; Figure  8D). We used these two genotypes as backgrounds to search for kinases and phosphatases that in knockdown conditions can modify the wing size phenotypes resulting from altered InR signaling. As a preliminary experiment, we tested whether known components of the InR pathway can modify the characteristic InR^DN^ or InR^*Act*^ wing phenotypes (Figure  8, E–H). We found that loss of *Akt*, *Pdk1*, *InR*, *Tor*, and *PI3K* consistently enhance the wing size and cell size defects caused by InR^DN^ expression (Figure  8G). The same knockdowns also significantly correct the larger than normal wing and cell size caused by expression of activated InR (Figure  8H). The examples of *Akt-RNAi and Pdk-RNAi* are shown in Figure  8, I–K and M–O, respectively. We also measured wing size for a collection of *UAS-RNAi* lines corresponding to genes that were identified under the dissecting microscope as “neutral” regarding InR^DN^ or InR^*Act*^ effects on wing size. In all cases, we could not find quantitative differences in the size of the corresponding combinations (Figure  8, E and F).

### Modifier screen of kinases and phosphatases in *InR* mutant backgrounds

**Figure 9:**
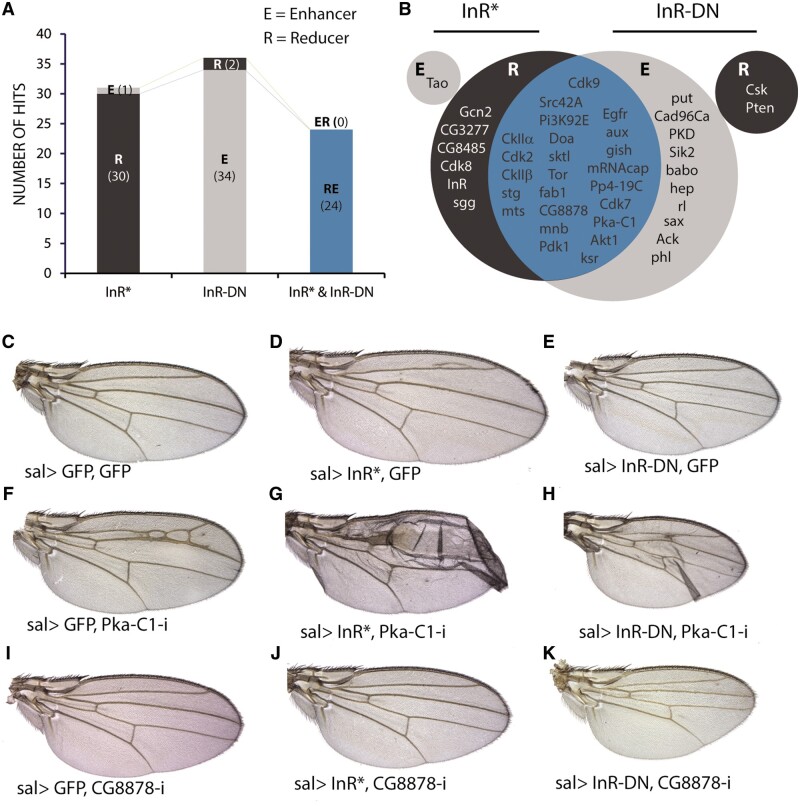
Modifications of InR^DN^ and InR^act^ phenotypes by knockdown of kinases and phosphatases. (A) Number of genes that behave as enhancers (gray section) or suppressors (black section) in the *sal^EPv^-Gal4 UAS-InR^Act^/UAS-RNAi* (InR*) and *sal^EPv^-Gal4 UAS-InR^DN^/UAS-RNAi* (InR-DN) genetic backgrounds. The blue column represents the number of genes that were simultaneously identified as suppressors of *sal^EPv^-Gal4 UAS-InR^Act^/UAS-RNAi* and enhancers of *sal^EPv^-Gal4 UAS-InR^DN^/UAS-RNAi.* (B) Genes identified in both *InR^Act^ and InR^DN^* screens as enhancers (gray) or suppressors (black). The overlap is colored in blue. (C–E) Control wings of *sal^EPv^-Gal4 UAS-GFP/UAS-GFP* (C), *sal^EPv^-Gal4 UAS-GFP/UAS-InR^Act^* (D), and *sal^EPv^-Gal4 UAS-GFP/UAS-InR^DN^* (E) genotype. (F–H) Example of UAS-PkaC1-RNAi on its own (F) and in combination with *UAS-InR^Act^* (G) and *UAS-InR^DN^* (H). (I–K) Example of UAS-CG8878-RNAi on its own (I) and in combination with *UAS-InR^Act^* (J) and *UAS-InR^DN^* (K).

**Figure 10:**
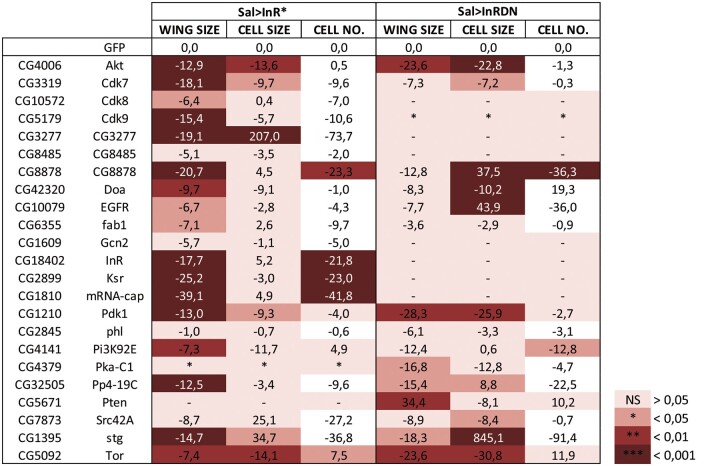
Numerical analysis of gene knockdowns modifying the wing size of InR^DN^ and InR^Act^ genetic combinations. Percentage of change in wing size, cell size, and estimated cell number (CELL NO.) of mutant combinations between *sal^EPv^-Gal4 UAS-InR^Act^* (Sal>InR*) or *sal^EPv^-Gal4 UAS-InR^DN^* (Sal>InRDN) and UAS-RNAi lines of genes modifying the corresponding values of *sal^EPv^-Gal4 UAS-InR^Act^/UAS-GFP* (GFP) or *sal^EPv^-Gal4 UAS-InR^DN^/UAS-GFP* control flies. Color code indicates the robustness of the change by the significance level.

We combined the collection of UAS-RNAi lines directed against protein kinases and phosphatases to generate *sal^EPv^-Gal4 UAS-In^Act^/UAS-RNAi and sal^EPv^-Gal4 UAS-InR^DN^/UAS-RNAi* flies, and selected those with wing sizes distinct to the corresponding *sal^EPv^-Gal4 UAS-InR^Act^/UAS-GFP and sal^EPv^-Gal4 UAS-InR^DN^/UAS-GFP* background phenotypes. We only found one enhancer of the InR^Act^ phenotype (*Tao*) and two suppressors of the InR^DN^ phenotype (*Csk and Pten*). In contrast, we found 30 suppressors of the InR^Act^ phenotype and 34 enhancers of the InR^DN^ phenotype (Figure  9A). Interestingly, 24 of these genes modify the InR^Act^ and InR^DN^ phenotypes in opposite manners, indicating that our screen has the potential to identify genes with a direct connection with Insulin signaling. In fact, we identified as “positive regulators” of InR signaling several known components of the pathway (*InR*, *Tor*, *Pdk1*, *Akt1*, and *PI3K92E*; Figures  9B and 10) and *Cadherin 96Ca* (*Cad96Ca*), encoding a receptor tyrosine kinase that cooperates with the InR during wing growth (O’Farrell *et al.* 2013). Other members of signaling pathways related to growth control identified in the screen were *Src42A*, *ksr*, *EGFR*, *rl*, and *phl* (EGFR signaling), the Hippo pathway member *Activated Cdc42 kinase* (*Ack*; Hu *et al.* 2016), and the TGFβ pathway components *punt*, *babo*, and *sax* (Figure  9B). We also identified as “positive regulators” of InR signaling several Cyclin-dependent kinases (Figures  9B and 10), including Cdk2, regulating G1, and S phases of the cell cycle, Cdk7, a component of the Cdk activating kinase complex with a function in promoting tissue growth through Yorki stabilization (Cho *et al.* 2020), Cdk9, involved in RNA polymerase II elongation control (Eissenberg *et al.* 2007), and Cdk8, a component of the Mediator complex (Loncle *et al.* 2007) that also participates in lipid homeostasis (Zhao *et al.* 2012). Other genes related to lipid metabolism were *Salt-inducible kinase 2* (*Sik2*), encoding a serine/threonine kinase that regulates lipid storage and energy homeostasis (Hirabayashi and Cagan 2015), and the regulatory (CkIIβ) and catalytic (CkIIα) subunits of the CKII (Figure  8B). Casein kinase II is a broad specificity Ser-Thr kinase involved in a variety of processes including cell signaling, neuronal physiology, transcription factor activity, and lipid and polyamine metabolism (Stark *et al.* 2011; Bandyopadhyay *et al.* 2016; McMillan *et al.* 2018). Gcn2, related to the regulation of amino acid metabolism (Kang *et al.* 2017) and translation initiation (Olsen *et al.* 1998) was identified as suppressor of the InR^Act^ large size phenotype (Figure  8B). Other genes identified in the screen as positive regulators of InR signaling encode proteins involved in vesicular trafficking such as *fab1 kinase* (*fab1*), encoding a phosphatidylinositol-3-phosphate 5-kinase promoting endosome-to lysosome trafficking (Rusten *et al.* 2006), *gilgamesh* (*gish*), encoding a plasma membrane-associated kinase regulating Rab11-mediated vesicle trafficking (Gault *et al.* 2012) and *auxilin* (*aux*), encoding a cofactor for the ATPase Hsc70 that regulates Clathrin dynamics (Kandachar *et al.* 2008). Finally, we also identified several genes regulating actin or tubulin dynamics, including *microtubule star* (*mts*), encoding the catalytic subunit of protein phosphatase 2A, *Protein Kinase D* (*PKD*), and the Phosphatidylinositol 4-Phosphate-5 kinase *skittles* (Gervais *et al.* 2008). Other kinases acting as positive regulators of InR signaling were CG8485 (fly ortholog of human SNF-related kinase), CG8878 (fly ortholog of VRK serine/threonine kinase 3; Figure  9, I–K), CG3277 (fly ortholog of human Colony-stimulating factor 1 receptor), Darkener of apricot (Doa), and *minibrain* (*mnb*).

## Concluding remarks

We used the *Drosophila* wing to identify the *in vivo* requirements of the *Drosophila* complement of kinases and phosphatases. Only a low percentage of Carbohydrate, Lipid, and Nucleoside kinases and phosphatases (29%) are required for the correct development of the wing. In contrast a higher percentage of protein kinases, phosphatidylinositol lipid phosphatases, cytoplasmic tyrosine phosphatases, and DSP are required for wing development (45–60% of genes). One caveat of our screen is that we used only one UAS-RNAi line per gene, and this can lead to a wrong estimation of phenotypic frequencies. However, the high coincidence of genes showing a wing phenotype (82%) identified in our screen and in a similar screen in which several independent lines were used suggests that the numbers of false positives and negatives are low. The most frequent phenotypes we observed for these genes were lethality and changes in the size of the wing, associated or not to changes in the position of the veins. These phenotypes are caused by changes in cell division, cell size, and cell viability. We also carried out several modifying screens aiming to identify protein kinases and phosphatases acting as regulators of the EGFR and InR signaling pathways. The correct activation of these pathways is a requisite for the growth and differentiation of the imaginal epithelium, and alterations on the level of their activities led to characteristic adult wing phenotypes that were used as sensitized backgrounds for these screens. We identified modifiers affecting one (11 genes) or more than one (24 genes) EGFR genetic background phenotypes, with genes acting as enhancers of EGFR gain of activity conditions usually behaving as suppressors of EGFR knockdown conditions and *vice versa*. We also identified a significant group of genes acting as enhancers of InR^DN^ and/or suppressors of InR^Act^ expression. These genes include kinases and phosphatases regulating lipid and amino acid metabolism, cytoskeleton dynamics and vesicle trafficking, other signaling pathways regulating wing growth and several Cyclin-dependent kinases such as Cdk2, Cdk7, Cdk8, and Cdk9 with a variety of functions in cell cycle regulation, tissue growth, RNA polymerase II elongation, and transcription.

## Data availability

The data underlying this article are available in the article and in its online supplementary material.

Supplementary material is available at *G3* online.

## Supplementary Material

jkab348_Supplementary_Data
