# Supplementary material for: Functional requirements of protein kinases and phosphatases in the development of the Drosophila melanogaster wing
Source: G3 (Bethesda). 2021 Oct 2;11(12):jkab348. doi: 10.1093/g3journal/jkab348 (PMC8664455; doi:10.1093/g3journal/jkab348)
Supplement: jkab348_Supplementary_Data [file jkab348_supplementary_data.zip › GENETICS-G3-2021-402588-s01.docx]

Acharya, J. K., Labarca, P., Delgado, R., Jalink, K., & Zuker, C. S. (1998). Synaptic defects and compensatory regulation of inositol metabolism in inositol polyphosphate 1-phosphatase mutants. *Neuron*, *20*(6), 1219–1229. https://doi.org/10.1016/S0896-6273(00)80502-4

Aihara, H., Nakagawa, T., Yasui, K., Ohta, T., Hirose, S., Dhomae, N., Takio, K., Kaneko, M., Takeshima, Y., Muramatsu, M., & Ito, T. (2004). Nucleosomal histone kinase-1 phosphorylates H2A Thr 119 during mitosis in the early Drosophila embryo. *Genes and Development*, *18*(8), 877–888. https://doi.org/10.1101/gad.1184604

Allen, A. M., Anreiter, I., Neville, M. C., & Sokolowski, M. B. (2017). Feeding-related traits are affected by dosage of the foraging gene in Drosophila melanogaster. *Geneticsn*, *205*(2), 761–773. https://doi.org/10.1534/genetics.116.197939

Alphey, L., Jimenez, J., White-Cooper, H., Dawson, I., Nurse, P., & Glover, D. M. (1992). twine, a cdc25 homolog that functions in the male and female germline of drosophila. *Cell*, *69*(6), 977–988. https://doi.org/10.1016/0092-8674(92)90616-K

Ambrosio, L., Mahowald, A. P., & Perrimon, N. (1989). Requirement of the Drosophila raf homologue for torso function. *Nature*, *342*(6247), 288–291. https://doi.org/10.1038/342288a0

Anamika, K., Abhinandan, K. R., Deshmukh, K., & Srinivasan, N. (2009). Classification of Nonenzymatic Homologues of Protein Kinases. *Comparative and Functional Genomics*, *2009*, 365637. https://doi.org/10.1155/2009/365637

Archambault, V., Zhao, X., White-Cooper, H., Carpenter, A. T. C., & Glover, D. M. (2007). Mutations in Drosophila Greatwall/Scant Reveal Its Roles in Mitosis and Meiosis and Interdependence with Polo Kinase. *PLoS Genetics*, *3*(11), e200. https://doi.org/10.1371/journal.pgen.0030200

Ashton-Beaucage, D., Udell, C. M., Gendron, P., Sahmi, M., Lefrançois, M., Baril, C., Guenier, A.-S., Duchaine, J., Lamarre, D., Lemieux, S., & Therrien, M. (2014). A Functional Screen Reveals an Extensive Layer of Transcriptional and Splicing Control Underlying RAS/MAPK Signaling in Drosophila. *PLoS Biology*, *12*(3), e1001809. https://doi.org/10.1371/journal.pbio.1001809

Baas, S., Sharrow, M., Kotu, V., Middleton, M., Nguyen, K., Flanagan-Steet, H., Aoki, K., & Tiemeyer, M. (2011). Sugar-free frosting, a homolog of SAD kinase, drives neural-specific glycan expression in the &lt;em&gt;Drosophila&lt;/em&gt; embryo. *Development*, *138*(3), 553 LP – 563. https://doi.org/10.1242/dev.055376

Balakrishnan, S. S., Basu, U., & Raghu, P. (2015). Phosphoinositide signalling in Drosophila. *Biochimica et Biophysica Acta (BBA) - Molecular and Cell Biology of Lipids*, *1851*(6), 770–784. https://doi.org/https://doi.org/10.1016/j.bbalip.2014.10.010

Bao, X., Deng, H., Johansen, J., Girton, J., & Johansen, K. M. (2007). Loss-of-Function Alleles of the JIL-1 Histone H3S10 Kinase Enhance Position-Effect Variegation at Pericentric Sites in Drosophila Heterochromatin. *Genetics*, *176*(2), 1355 LP – 1358. https://doi.org/10.1534/genetics.107.073676

Baril, C., Sahmi, M., Ashton-Beaucage, D., Stronach, B., & Therrien, M. (2009). The PP2C alphabet is a negative regulator of stress-activated protein kinase signaling in Drosophila. *Genetics*, *181*(2), 567–579. https://doi.org/10.1534/genetics.108.096461

Bartkowiak, B., Liu, P., Phatnani, H. P., Fuda, N. J., Cooper, J. J., Price, D. H., Adelman, K., Lis, J. T., & Greenleaf, A. L. (2010). CDK12 is a transcription elongation-associated CTD kinase, the metazoan ortholog of yeast Ctk1. *Genes and Development*, *24*(20), 2303–2316. https://doi.org/10.1101/gad.1968210

Beckstead, R. B., Ner, S. S., Hales, K. G., Grigliatti, T. A., Baker, B. S., & Bellen, H. J. (2005). Bonus, a Drosophila TIF1 Homolog, Is a Chromatin-Associated Protein That Acts as a Modifier of Position-Effect Variegation. *Genetics*, *169*(2), 783 LP – 794. https://doi.org/10.1534/genetics.104.037085

Betson, M., & Settleman, J. (2007). A Rho-binding protein kinase C-like activity is required for the function of protein kinase N in drosophila development. *Genetics*, *176*(4), 2201–2212. https://doi.org/10.1534/genetics.107.072967

Bilak, A., Uyetake, L., & Su, T. T. (2014). Dying Cells Protect Survivors from Radiation-Induced Cell Death in Drosophila. *PLoS Genetics*, *10*(3), e1004220. https://doi.org/10.1371/journal.pgen.1004220

Binari, R., & Perrimon, N. (1994). Stripe-specific regulation of pair-rule genes by hopscotch, a putative Jak family tyrosine kinase in Drosophila. *Genes & Development* , *8*(3), 300–312. https://doi.org/10.1101/gad.8.3.300

Bonini, N. M., Leiserson, W. M., & Benzer, S. (1993). The eyes absent gene: Genetic control of cell survival and differentiation in the developing Drosophila eye. *Cell*, *72*(3), 379–395. https://doi.org/10.1016/0092-8674(93)90115-7

Braden, C. R., & Neufeld, T. P. (2016). Atg1-independent induction of autophagy by the Drosophila Ulk3 homolog, ADUK. *The FEBS Journal*, *283*(21), 3889–3897. https://doi.org/https://doi.org/10.1111/febs.13906

Britton, J. S., Lockwood, W. K., Li, L., Cohen, S. M., & Edgar, B. A. (2002). Drosophila’s insulin/PI3-kinase pathway coordinates cellular metabolism with nutritional conditions. *Developmental Cell*, *2*(2), 239–249. https://doi.org/10.1016/S1534-5807(02)00117-X

Brodsky, M. H., & Steller, H. (1996). Positional Information along the Dorsal–Ventral Axis of theDrosophilaEye: Graded Expression of thefour-jointedGene. *Developmental Biology*, *173*(2), 428–446. https://doi.org/https://doi.org/10.1006/dbio.1996.0038

Brodsky, M. H., Sekelsky, J. J., Tsang, G., Hawley, R. S., & Rubin, G. M. (2000). mus304 encodes a novel DNA damage checkpoint protein required during Drosophila development. *Genes and Development*, *14*(6), 666–678. https://doi.org/10.1101/gad.14.6.666

Brooks, D., Naeem, F., Stetsiv, M., Goetting, S. C., Bawa, S., Green, N., Clark, C., Bashirullah, A., & Geisbrecht, E. R. (2020). Drosophila NUAK functions with Starvin/BAG3 in autophagic protein turnover. *PLoS Genetics*, *16*(4), e1008700. https://doi.org/10.1371/journal.pgen.1008700

Brownlee, C. W., Klebba, J. E., Buster, D. W., & Rogers, G. C. (2011). The protein phosphatase 2A regulatory subunit Twins stabilizes Plk4 to induce centriole amplification. *Journal of Cell Biology*, *195*(2), 231–243. https://doi.org/10.1083/jcb.201107086

Brummel, T., Abdollah, S., Haerry, T. E., Shimell, M. J., Merriam, J., Raftery, L., Wrana, J. L., & O’Connor, M. B. (1999). The Drosophila activin receptor Baboon signals through dSmad2 and controls cell proliferation but not patterning during larval development. *Genes and Development*, *13*(1), 98–111. <https://doi.org/10.1101/gad.13.1.98>

Bunker, B. D., Nellimoottil, T. T., Boileau, R. M., Classen, A. K., & Bilder, D. (2015). The transcriptional response to tumorigenic polarity loss in Drosophila. *ELife*, *2015*(4). https://doi.org/10.7554/eLife.03189

Burgess, J., Del Bel, L. M., Ma, C. I. J., Barylko, B., Polevoy, G., Rollins, J., Albanesi, J. P., Krämer, H., & Brill, J. A. (2012). Type II phosphatidylinositol 4-kinase regulates trafficking of secretory granule proteins in Drosophila. *Development (Cambridge)*, *139*(16), 3040–3050. https://doi.org/10.1242/dev.077644

Buschmann, J., Moritz, B., Jeske, M., Lilie, H., Schierhorn, A., & Wahle, E. (2013). Identification of drosophila and human 7-methyl GMP-specific nucleotidases. *Journal of Biological Chemistry*, *288*(4), 2441–2451. https://doi.org/10.1074/jbc.M112.426700

Campbell, S. D., Sprenger, F., Edgar, B. A., & O’Farrell, P. H. (1995). Drosophila Wee1 kinase rescues fission yeast from mitotic catastrophe and phosphorylates Drosophila Cdc2 in vitro. *Molecular Biology of the Cell*, *6*(10), 1333–1347. <https://doi.org/10.1091/mbc.6.10.1333>

Campos, I., Geiger, J. A., Santos, A. C., Carlos, V., & Jacinto, A. (2010). Genetic Screen in Drosophila melanogasterUncovers a Novel Set of Genes Required for Embryonic Epithelial Repair. *Genetics*, *184*(1), 129 LP – 140. https://doi.org/10.1534/genetics.109.110288

Cao, J., Li, Y., Xia, W., Reddig, K., Hu, W., Xie, W., Li, H.-S., & Han, J. (2011). A Drosophila metallophosphoesterase mediates deglycosylation of rhodopsin. *The EMBO Journal*, *30*(18), 3701–3713. https://doi.org/https://doi.org/10.1038/emboj.2011.254

Carim, S. C., Ben El Kadhi, K., Yan, G., Sweeney, S. T., Hickson, G. R., Carréno, S., & Lowe, M. (2019). IPIP27 Coordinates PtdIns(4,5)P 2 Homeostasis for Successful Cytokinesis. *Current Biology*, *29*(5), 775-789.e7. https://doi.org/10.1016/j.cub.2019.01.043

Carmena, M., Riparbelli, M. G., Minestrini, G., Tavares, Á. M., Adams, R., Callaini, G., & Glover, D. M. (1998). Drosophila polo kinase is required for cytokinesis. *Journal of Cell Biology*, *143*(3), 659–671. https://doi.org/10.1083/jcb.143.3.659

Carrera, P., Moshkin, Y. M., Grönke, S., Silljé, H. H. W., Nigg, E. A., Jäckle, H., & Karch, F. (2003). Tousled-like kinase functions with the chromatin assembly pathway regulating nuclear divisions. *Genes and Development*, *17*(20), 2578–2590. https://doi.org/10.1101/gad.276703

Chakrabarti, S., Poidevin, M., & Lemaitre, B. (2014). The Drosophila MAPK p38c Regulates Oxidative Stress and Lipid Homeostasis in the Intestine. *PLoS Genetics*, *10*(9), e1004659. https://doi.org/10.1371/journal.pgen.1004659

Champagne, M. B., Edwards, K. A., Erickson, H. P., & Kiehart, D. P. (2000). Drosophila Stretchin-MLCK is a Novel Member of the Titin/Myosin Light Chain Kinase Family. *Journal of Molecular Biology*, *300*(4), 759–777. https://doi.org/https://doi.org/10.1006/jmbi.2000.3802

Chandrasekaran, V., & Beckendorf, S. K. (2005). Tec29 controls actin remodelling and endoreplication during invagination of the Drosophila embryonic salivary glands. *Development*, *132*(15), 3515–3524. https://doi.org/10.1242/dev.01926

Chen, F., Archambault, V., Kar, A., Lio’, P., D’Avino, P. P., Sinka, R., Lilley, K., Laue, E. D., Deak, P., Capalbo, L., & Glover, D. M. (2007). Multiple Protein Phosphatases Are Required for Mitosis in Drosophila. *Current Biology*, *17*(4), 293–303. https://doi.org/10.1016/j.cub.2007.01.068

Chen, G.-C., Gajowniczek, P., & Settleman, J. (2004). Rho-LIM Kinase Signaling Regulates Ecdysone-Induced Gene Expression and Morphogenesis during Drosophila Metamorphosis. *Current Biology*, *14*(4), 309–313. https://doi.org/10.1016/j.cub.2004.01.056

Chen, J., Ezzeddine, N., Waltenspiel, B., Albrecht, T. R., Warren, W. D., Marzluff, W. F., & Wagner, E. J. (2012). An RNAi screen identifies additional members of the Drosophila Integrator complex and a requirement for cyclin C/Cdk8 in snRNA 3′-end formation. *RNA*, *18*(12), 2148–2156. https://doi.org/10.1261/rna.035725.112

Chen, P., Zhou, Z., Yao, X., Pang, S., Liu, M., Jiang, W., Jiang, J., & Zhang, Q. (2017). Capping Enzyme mRNA-cap/RNGTT Regulates Hedgehog Pathway Activity by Antagonizing Protein Kinase A. *Scientific Reports*, *7*(1), 2891. https://doi.org/10.1038/s41598-017-03165-2

Chen, X., Oh, S. W., Zheng, Z., Chen, H. W., Shin, H. H., & Hou, S. S. (2003). Cyclin D-Cdk4 and cyclin E-Cdk2 regulate the JAK/STAT signal transduction pathway in Drosophila. *Developmental Cell*, *4*(2), 179–190. https://doi.org/10.1016/S1534-5807(03)00024-8

Cheng, S., Maier, D., Neubueser, D., & Hipfner, D. R. (2010). Regulation of Smoothened by Drosophila G-protein-coupled receptor kinases. *Developmental Biology*, *337*(1), 99–109. https://doi.org/https://doi.org/10.1016/j.ydbio.2009.10.014

Cheng, Y. L. L., & Andrew, D. J. J. (2015). Extracellular Mipp1 Activity Confers Migratory Advantage to Epithelial Cells during Collective Migration. *Cell Reports*, *13*(10), 2174–2188. https://doi.org/10.1016/j.celrep.2015.10.071

Chi, C., Wang, L., Lan, W., Zhao, L., & Su, Y. (2018). PpV, acting via the JNK pathway, represses apoptosis during normal development of Drosophila wing. *Apoptosis*, *23*(9–10), 554–562. <https://doi.org/10.1007/s10495-018-1479-2>

Chien, C.T., Wang, S., Rothenberg, M., Jan, L.Y., Jan, Y.N. (1998). Numb-associated kinase interacts with the phosphotyrosine binding domain of Numb and antagonizes the function of Numb in vivo.  Mol. Cell. Biol. 18(1): 598--607.

Cho, K. S., Lee, J. H., Kim, S., Kim, D., Koh, H., Lee, J., Kim, C., Kim, J., & Chung, J. (2001). Drosophila phosphoinositide-dependent kinase-1 regulates apoptosis and growth via the phosphoinositide 3-kinase-dependent signaling pathway. *Proceedings of the National Academy of Sciences of the United States of America*, *98*(11), 6144–6149. https://doi.org/10.1073/pnas.101596998

Choi, S., Kim, W., & Chung, J. (2011). Drosophila salt-inducible kinase (SIK) regulates starvation resistance through cAMP-response element-binding protein (CREB)-regulated transcription coactivator (CRTC). *The Journal of Biological Chemistry*, *286*(4), 2658–2664. https://doi.org/10.1074/jbc.C110.119222

Choi, S., Lim, D.-S., & Chung, J. (2015). Feeding and Fasting Signals Converge on the LKB1-SIK3 Pathway to Regulate Lipid Metabolism in Drosophila. *PLOS Genetics*, *11*(5), e1005263. https://doi.org/10.1371/journal.pgen.1005263

Claret, S., Jouette, J., Benoit, B., Legent, K., & Guichet, A. (2014). PI(4,5)P2 produced by the PI4P5K SKTL controls apical size by tethering PAR-3 in drosophila epithelial cells. *Current Biology*, *24*(10), 1071–1079. https://doi.org/10.1016/j.cub.2014.03.056

Conder, R., Yu, H., Ricos, M., Hing, H., Chia, W., Lim, L., & Harden, N. (2004). dPak is required for integrity of the leading edge cytoskeleton during Drosophila dorsal closure but does not signal through the JNK cascade. *Developmental Biology*, *276*(2), 378–390. https://doi.org/https://doi.org/10.1016/j.ydbio.2004.08.044

Connell-Crowley, L., Le Gall, M., Vo, D. J., & Giniger, E. (2000). The cyclin-dependent kinase Cdk5 controls multiple aspects of axon patterning in vivo. *Current Biology*, *10*(10), 599–603. https://doi.org/10.1016/S0960-9822(00)00487-5

Cronin, S. J., Nehme, N. T., Limmer, S., Liegeois, S., Pospisilik, J. A., Schramek, D., Leibbrandt, A., Simoes Rde, M., Gruber, S., Puc, U., Ebersberger, I., Zoranovic, T., Neely, G. G., von Haeseler, A., Ferrandon, D., & Penninger, J. M. (2009). Genome-wide RNAi screen identifies genes involved in intestinal pathogenic bacterial infection. *Science*, *325*(5938), 340–343. https://doi.org/10.1126/science.1173164

Cully, M., Genevet, A., Warne, P., Treins, C., Liu, T., Bastien, J., Baum, B., Tapon, N., Leevers, S. J., & Downward, J. (2010). A Role for p38 Stress-Activated Protein Kinase in Regulation of Cell Growth via TORC1. *Molecular and Cellular Biology*, *30*(2), 481–495. <https://doi.org/10.1128/mcb.00688-09>

Das, R., Sebo, Z., Pence, L., & Dobens, L. L. (2014). Drosophila Tribbles Antagonizes Insulin Signaling-Mediated Growth and Metabolism via Interactions with Akt Kinase. *PLoS ONE*, *9*(10), e109530. https://doi.org/10.1371/journal.pone.0109530

Dean, D. M., Maroja, L. S., Cottrill, S., Bomkamp, B. E., Westervelt, K. A., & Deitcher, D. L. (2016). The &lt;em&gt;wavy&lt;/em&gt; Mutation Maps to the &lt;em&gt;Inositol 1,4,5-Trisphosphate 3-Kinase 2&lt;/em&gt; (&lt;em&gt;IP3K2&lt;/em&gt;) Gene of &lt;em&gt;Drosophila&lt;/em&gt; and Interacts with &lt;em&gt;IP3R&lt;/em&gt; to Affect Wing Development. *G3: Genes|Genomes|Genetics*, *6*(2), 299 LP – 310. https://doi.org/10.1534/g3.115.024307

Dearborn, R. E., Dai, Y., Reed, B., Karian, T., Gray, J., & Kunes, S. (2012). Reph, a Regulator of Eph Receptor Expression in the Drosophila melanogaster Optic Lobe. *PLoS ONE*, *7*(5), e37303. https://doi.org/10.1371/journal.pone.0037303

Degoutin, J. L., Milton, C. C., Yu, E., Tipping, M., Bosveld, F., Yang, L., Bellaiche, Y., Veraksa, A., & Harvey, K. F. (2013). Riquiqui and Minibrain are regulators of the Hippo pathway downstream of Dachsous. *Nature Cell Biology*, *15*(10), 1176–1185. https://doi.org/10.1038/ncb2829

Desai, C. J., Gindhart, J. G., Goldstein, L. S. B., & Zinn, K. (1996). Receptor tyrosine phosphatases are required for motor axon guidance in the Drosophila embryo. *Cell*, *84*(4), 599–609. https://doi.org/10.1016/S0092-8674(00)81035-1

Dijkers, P. F., & O’Farrell, P. H. (2007). Drosophila Calcineurin Promotes Induction of Innate Immune Responses. *Current Biology*, *17*(23), 2087–2093. https://doi.org/10.1016/j.cub.2007.11.001

Dombrádi, V., & Cohen, P. T. W. (1992). Protein phosphorylation is involved in the regulation of chromatin condensation during interphase. *FEBS Letters*, *312*(1), 21–26. https://doi.org/https://doi.org/10.1016/0014-5793(92)81402-8

Ducat, D., Kawaguchi, S., Liu, H., Yates, J. R., & Zheng, Y. (2008). Regulation of Microtubule Assembly and Organization in Mitosis by the AAA+ ATPase Pontin. *Molecular Biology of the Cell*, *19*(7), 3097–3110. https://doi.org/10.1091/mbc.e07-11-1202

Edgar, B. A., & O’Farrell, P. H. (1990). The three postblastoderm cell cycles of Drosophila embryogenesis are regulated in G2 by string. *Cell*, *62*(3), 469–480. https://doi.org/10.1016/0092-8674(90)90012-4

Eissenberg, J. C., Shilatifard, A., Dorokhov, N., & Michener, D. E. (2007). Cdk9 is an essential kinase in Drosophila that is required for heat shock gene expression, histone methylation and elongation factor recruitment. *Molecular Genetics and Genomics*, *277*(2), 101–114. https://doi.org/10.1007/s00438-006-0164-2

Ertürk-Hasdemir, D., Broemer, M., Leulier, F., Lane, W. S., Paquette, N., Hwang, D., Kim, C.-H., Stöven, S., Meier, P., & Silverman, N. (2009). Two roles for the &lt;em&gt;Drosophila&lt;/em&gt; IKK complex in the activation of Relish and the induction of antimicrobial peptide genes. *Proceedings of the National Academy of Sciences*, *106*(24), 9779 LP – 9784. https://doi.org/10.1073/pnas.0812022106

Fenger, D. D., Carminati, J. L., Burney-Sigman, D. L., Kashevsky, H., Dines, J. L., Elfring, L. K., & Orr-Weaver, T. L. (2000). PAN GU: a protein kinase that inhibits S phase and promotes mitosis in early Drosophila development. *Development*, *127*(22).

Fenckova, M., Hobizalova, R., Fric, Z. F., & Dolezal, T. (2011). Functional characterization of ecto-5′-nucleotidases and apyrases in Drosophila melanogaster. *Insect Biochemistry and Molecular Biology*, *41*(12), 956–967. https://doi.org/https://doi.org/10.1016/j.ibmb.2011.09.005

Fernandez, R., Takahashi, F., Liu, Z., Steward, R., Stein, D., & Stanley, E. R. (2000). The Drosophila Shark tyrosine kinase is required for embryonic dorsal closure. *Genes & Development* , *14*(5), 604–614. <https://doi.org/10.1101/gad.14.5.604>

Findlay, G. D., MacCoss, M. J., & Swanson, W. J. (2009). Proteomic discovery of previously unannotated, rapidly evolving seminal fluid genes in Drosophila. *Genome Research* , *19*(5), 886–896. https://doi.org/10.1101/gr.089391.108

Forrest, S., Chai, A., Sanhueza, M., Marescotti, M., Parry, K., Georgiev, A., Sahota, V., Mendez-Castro, R., & Pennetta, G. (2013). Increased levels of phosphoinositides cause neurodegeneration in a Drosophila model of amyotrophic lateral sclerosis. *Human Molecular Genetics*, *22*(13), 2689–2704. https://doi.org/10.1093/hmg/ddt118

Fresán, U., Rodríguez-Sánchez, M. A., Reina, O., Corces, V. G., & Lluisa Espinàs, M. (2020). Haspin kinase modulates nuclear architecture and Polycomb-dependent gene silencing. *PLoS Genetics*, *16*(8), e1008962. https://doi.org/10.1371/JOURNAL.PGEN.1008962

Frolov, M. V., Benevolenskaya, E. V., & Birchler, J. A. (2001). Molecular analysis of a novel Drosophila diacylglycerol kinase, DGKε. *Biochimica et Biophysica Acta - Molecular Cell Research*, *1538*(2–3), 339–352. https://doi.org/10.1016/S0167-4889(01)00085-4

Fuhrmann, J., Mierzwa, B., Trentini, D. B., Spiess, S., Lehner, A., Charpentier, E., & Clausen, T. (2013). Structural Basis for Recognizing Phosphoarginine and Evolving Residue-Specific Protein Phosphatases in Gram-Positive Bacteria. *Cell Reports*, *3*(6), 1832–1839. https://doi.org/10.1016/j.celrep.2013.05.023

Fukumoto, T., Watanabe-Fukunaga, R., Fujisawa, K., Nagata, S., & Fukunaga, R. (2001). The Fused Protein Kinase Regulates Hedgehog-stimulated Transcriptional Activation in Drosophila Schneider 2 Cells . *Journal of Biological Chemistry* , *276*(42), 38441–38448. https://doi.org/10.1074/jbc.M105871200

Furlong, E. E. M., Andersen, E. C., Null, B., White, K. P., & Scott, M. P. (2001). Patterns of Gene Expression During &lt;em&gt;Drosophila &lt;/em&gt;Mesoderm Development. *Science*, *293*(5535), 1629 LP – 1633. https://doi.org/10.1126/science.1062660

Gao, T., Furnari, F., & Newton, A. C. (2005). PHLPP: A phosphatase that directly dephosphorylates Akt, promotes apoptosis, and suppresses tumor growth. *Molecular Cell*, *18*(1), 13–24. https://doi.org/10.1016/j.molcel.2005.03.008

Garcia-Murillas, I., Pettitt, T., Macdonald, E., Okkenhaug, H., Georgiev, P., Trivedi, D., Hassan, B., Wakelam, M., & Raghu, P. (2006). lazaro encodes a lipid phosphate phosphohydrolase that regulates phosphatidylinositol turnover during Drosophila phototransduction. *Neuron*, *49*(4), 533–546. https://doi.org/10.1016/j.neuron.2006.02.001

Gaudet, P., Livstone, M. S., Lewis, S. E., & Thomas, P. D. (2011). Phylogenetic-based propagation of functional annotations within the Gene Ontology consortium. *Briefings in Bioinformatics*, *12*(5), 449–462. https://doi.org/10.1093/bib/bbr042

Geng, W., He, B., Wang, M., & Adler, P. N. (2000). The tricornered Gene, Which Is Required for the Integrity of Epidermal Cell Extensions, Encodes the Drosophila Nuclear DBF2-Related Kinase. *Genetics*, *156*(4), 1817 LP – 1828. http://www.genetics.org/content/156/4/1817.abstract

Geuking, P., Narasimamurthy, R., Lemaitre, B., Basler, K., & Leulier, F. (2009). A Non-Redundant Role for Drosophila Mkk4 and Hemipterous/Mkk7 in TAK1-Mediated Activation of JNK. *PLoS ONE*, *4*(11), e7709. https://doi.org/10.1371/journal.pone.0007709

Giet, R., & Glover, D. M. (2001). Drosophila aurora B kinase is required for histone H3 phosphorylation and condensin recruitment during chromosome condensation and to organize the central spindle during cytokinesis. *Journal of Cell Biology*, *152*(4), 669–681. https://doi.org/10.1083/jcb.152.4.669

Gillespie, J., & Hodge, J. (2013). CASK regulates CaMKII autophosphorylation in neuronal growth, calcium signaling, and learning  . In *Frontiers in Molecular Neuroscience* (Vol. 6, p. 27). https://www.frontiersin.org/article/10.3389/fnmol.2013.00027

Glover, D. M., Leibowitz, M. H., McLean, D. A., & Parry, H. (1995). Mutations in aurora prevent centrosome separation leading to the formation of monopolar spindles. *Cell*, *81*(1), 95–105. https://doi.org/10.1016/0092-8674(95)90374-7

Gluderer, S., Brunner, E., Germann, M., Jovaisaite, V., Li, C., Rentsch, C. A., Hafen, E., & Stocker, H. (2010). Madm (Mlf1 adapter molecule) cooperates with bunched a to promote growth in drosophila. *Journal of Biology*, *9*(1), 9. https://doi.org/10.1186/jbiol216

Godena, V. K., Brookes-Hocking, N., Moller, A., Shaw, G., Oswald, M., Sancho, R. M., Miller, C. C. J., Whitworth, A. J., & De Vos, K. J. (2014). Increasing microtubule acetylation rescues axonal transport and locomotor deficits caused by LRRK2 Roc-COR domain mutations. *Nature Communications*, *5*(1), 5245. https://doi.org/10.1038/ncomms6245

González-Mariscal, I., Martin-Montalvo, A., Vazquez-Fonseca, L., Pomares-Viciana, T., Sánchez-Cuesta, A., Fernández-Ayala, D. J., Navas, P., & Santos-Ocana, C. (2018). The mitochondrial phosphatase PPTC7 orchestrates mitochondrial metabolism regulating coenzyme Q10 biosynthesis. *Biochimica et Biophysica Acta (BBA) - Bioenergetics*, *1859*(11), 1235–1248. <https://doi.org/https://doi.org/10.1016/j.bbabio.2018.09.369>

Gorski, S. M., Chittaranjan, S., Pleasance, E. D., Freeman, J. D., Anderson, C. L., Varhol, R. J., Coughlin, S. M., Zuyderduyn, S. D., Jones, S. J. M., & Marra, M. A. (2003). A SAGE approach to discovery of genes involved in autophagic cell death. *Current Biology*, *13*(4), 358–363. <https://doi.org/10.1016/S0960-9822(03)00082-4>

Gouzi, J. Y., Moressis, A., Walker, J. A., Apostolopoulou, A. A., Palmer, R. H., Bernards, A., & Skoulakis, E. M. C. (2011). The Receptor Tyrosine Kinase Alk Controls Neurofibromin Functions in Drosophila Growth and Learning. *PLoS Genetics*, *7*(9), e1002281. https://doi.org/10.1371/journal.pgen.1002281

Greenspan, L., & Clark, A. G. (2011). Associations between Variation in X Chromosome Male Reproductive Genes and Sperm Competitive Ability in *Drosophila melanogaster*. *International Journal of Evolutionary Biology*, *2011*, 214280. https://doi.org/10.4061/2011/214280

Grevengoed, E. E., Loureiro, J. J., Jesse, T. L., & Peifer, M. (2001). Abelson kinase regulates epithelial morphogenesis in Drosophila . *Journal of Cell Biology*, *155*(7), 1185–1198. https://doi.org/10.1083/jcb.200105102

Guelman, S., Suganuma, T., Florens, L., Weake, V., Swanson, S. K., Washburn, M. P., Abmayr, S. M., & Workman, J. L. (2006). The Essential Gene &lt;em&gt;wda&lt;/em&gt; Encodes a WD40 Repeat Subunit of &lt;em&gt;Drosophila&lt;/em&gt; SAGA Required for Histone H3 Acetylation. *Molecular and Cellular Biology*, *26*(19), 7178 LP – 7189. https://doi.org/10.1128/MCB.00130-06

Guo, X., Engel, J. L., Xiao, J., Tagliabracci, V. S., Wang, X., Huang, L., & Dixon, J. E. (2011). UBLCP1 is a 26S proteasome phosphatase that regulates nuclear proteasome activity. *Proceedings of the National Academy of Sciences*, *108*(46), 18649 LP – 18654. https://doi.org/10.1073/pnas.1113170108

Gupta, A., Toscano, S., Trivedi, D., Jones, D. R., Mathre, S., Clarke, J. H., Divecha, N., & Raghu, P. (2013). Phosphatidylinositol 5-phosphate 4-kinase (PIP4K) regulates TOR signaling and cell growth during &lt;em&gt;Drosophila&lt;/em&gt; development. *Proceedings of the National Academy of Sciences*, *110*(15), 5963 LP – 5968. https://doi.org/10.1073/pnas.1219333110

Hagedorn, E. J., Bayraktar, J. L., Kandachar, V. R., Bai, T., Englert, D. M., & Chang, H. C. (2006). Drosophila melanogaster auxilin regulates the internalization of Delta to control activity of the Notch signaling pathway. *Journal of Cell Biology*, *173*(3), 443–452. https://doi.org/10.1083/jcb.200602054

Hain, D., Langlands, A., Sonnenberg, H. C., Bailey, C., Bullock, S. L., & Muller, H. A. J. (2014). The Drosophila MAST kinase drop out is required to initiate membrane compartmentalisation during cellularisation and regulates dynein-based transport. *Development (Cambridge)*, *141*(10), 2119–2130. https://doi.org/10.1242/dev.104711

Han, Z. S., Enslen, H., Hu, X., Meng, X., Wu, I.-H., Barrett, T., Davis, R. J., & Ip, Y. T. (1998). A Conserved p38 Mitogen-Activated Protein Kinase Pathway Regulates Drosophila Immunity Gene Expression. *Molecular and Cellular Biology*, *18*(6), 3527–3539. https://doi.org/10.1128/mcb.18.6.3527

Hatzihristidis, T., Desai, N., Hutchins, A. P., Meng, T. C., Tremblay, M. L., & Miranda-Saavedra, D. (2015). A Drosophila-centric view of protein tyrosine phosphatases. In *FEBS Letters* (Vol. 589, Issue 9, pp. 951–966). Elsevier B.V. https://doi.org/10.1016/j.febslet.2015.03.005

Helps, N. R., Brewis, N. D., Lineruth, K., Davis, T., Kaiser, K., & Cohen, P. T. (1998). Protein phosphatase 4 is an essential enzyme required for organisation of microtubules at centrosomes in Drosophila embryos. *Journal of Cell Science*, *111*(10).

Hennig, K. M., Colombani, J., & Neufeld, T. P. (2006). TOR coordinates bulk and targeted endocytosis in the Drosophila melanogaster fat body to regulate cell growth. *Journal of Cell Biology*, *173*(6), 963–974. https://doi.org/10.1083/jcb.200511140

Herold, N., Will, C. L., Wolf, E., Kastner, B., Urlaub, H., & Lührmann, R. (2009). Conservation of the Protein Composition and Electron Microscopy Structure of Drosophila melanogaster and Human Spliceosomal Complexes. *Molecular and Cellular Biology*, *29*(1), 281–301. https://doi.org/10.1128/mcb.01415-08

Herr, D. R., Fyrst, H., Creason, M. B., Phan, V. H., Saba, J. D., & Harris, G. L. (2004). Characterization of the Drosophila Sphingosine Kinases and Requirement for Sk2 in Normal Reproductive Function. *Journal of Biological Chemistry*, *279*(13), 12685–12694. https://doi.org/10.1074/JBC.M310647200

Hipfner, D. R., & Cohen, S. M. (2003). The Drosophila Sterile-20 Kinase Slik Controls Cell Proliferation and Apoptosis during Imaginal Disc Development. *PLoS Biology*, *1*(2), e35. https://doi.org/10.1371/journal.pbio.0000035

Huang, H.-R., Chen, Z. J., Kunes, S., Chang, G.-D., & Maniatis, T. (2010). Endocytic pathway is required for Drosophila Toll innate immune signaling. *Proceedings of the National Academy of Sciences*, *107*(18), 8322–8327. https://doi.org/10.1073/PNAS.1004031107

Huang, H.-L., Wang, S., Yin, M.-X., Dong, L., Wang, C., Wu, W., Lu, Y., Feng, M., Dai, C., Guo, X., Li, L., Zhao, B., Zhou, Z., Ji, H., Jiang, J., Zhao, Y., Liu, X.-Y., & Zhang, L. (2013). Par-1 Regulates Tissue Growth by Influencing Hippo Phosphorylation Status and Hippo-Salvador Association. *PLoS Biology*, *11*(8), e1001620. https://doi.org/10.1371/journal.pbio.1001620

Ibar, C., Cataldo, V. F., Vásquez-Doorman, C., Olguín, P., & Glavic, Á. (2013). &lt;em&gt;Drosophila&lt;/em&gt; p53-related protein kinase is required for PI3K/TOR pathway-dependent growth. *Development*, *140*(6), 1282 LP – 1291. https://doi.org/10.1242/dev.086918

Imai, Y., Kanao, T., Sawada, T., Kobayashi, Y., Moriwaki, Y., Ishida, Y., Takeda, K., Ichijo, H., Lu, B., & Takahashi, R. (2010). The Loss of PGAM5 Suppresses the Mitochondrial Degeneration Caused by Inactivation of PINK1 in Drosophila. *PLoS Genetics*, *6*(12), e1001229. https://doi.org/10.1371/journal.pgen.1001229

Inoue, H., Tateno, M., Fujimura-Kamada, K., Takaesu, G., Adachi-Yamada, T., Ninomiya-Tsuji, J., Irie, K., Nishida, Y., & Matsumoto, K. (2001). A Drosophila MAPKKK, D-MEKK1, mediates stress responses through activation of p38 MAPK. *EMBO Journal*, *20*(19), 5421–5430. https://doi.org/10.1093/emboj/20.19.5421

Ishikawa, H. O., Xu, A., Ogura, E., Manning, G., & Irvine, K. D. (2012). The Raine Syndrome Protein FAM20C Is a Golgi Kinase That Phosphorylates Bio-Mineralization Proteins. *PLoS ONE*, *7*(8), e42988. https://doi.org/10.1371/journal.pone.0042988

Ivanovska, I., Lee, E., Kwan, K. M., Fenger, D. D., & Orr-Weaver, T. L. (2004). The Drosophila MOS Ortholog Is Not Essential for Meiosis. *Current Biology*, *14*(1), 75–80. https://doi.org/10.1016/j.cub.2003.12.031

Jean, S., Cox, S., Schmidt, E. J., Robinson, F. L., & Kiger, A. (2012). Sbf/MTMR13 coordinates PI(3)P and Rab21 regulation in endocytic control of cellular remodeling. *Molecular Biology of the Cell*, *23*(14), 2723–2740. https://doi.org/10.1091/mbc.e12-05-0375

Jeon, M., Nguyen, H., Bahri, S., & Zinn, K. (2008). Redundancy and compensation in axon guidance: Genetic analysis of the Drosophila Ptp10D/Ptp4E receptor tyrosine phosphatase subfamily. *Neural Development*, *3*(1), 3. https://doi.org/10.1186/1749-8104-3-3

Jeon, M., & Zinn, K. (2009). Receptor tyrosine phosphatases control tracheal tube geometries through negative regulation of Egfr signaling. *Development*, *136*(18), 3121–3129. https://doi.org/10.1242/dev.033597

Jha, A. R., Zhou, D., Brown, C. D., Kreitman, M., Haddad, G. G., & White, K. P. (2016). Shared Genetic Signals of Hypoxia Adaptation in Drosophila and in High-Altitude Human Populations. *Molecular Biology and Evolution*, *33*(2), 501–517. https://doi.org/10.1093/molbev/msv248

Johnson Hamlet, M. R., & Perkins, L. A. (2001). Analysis of corkscrew signaling in the Drosophila epidermal growth factor receptor pathway during myogenesis. *Genetics*, *159*(3), 1073–1087. https://pubmed.ncbi.nlm.nih.gov/11729154

Johnson, E. L., Fetter, R. D., & Davis, G. W. (2009). Negative Regulation of Active Zone Assembly by a Newly Identified SR Protein Kinase. *PLoS Biology*, *7*(9), e1000193. https://doi.org/10.1371/journal.pbio.1000193

Juhász, G., Hill, J. H., Yan, Y., Sass, M., Baehrecke, E. H., Backer, J. M., & Neufeld, T. P. (2008). The class III PI(3)K Vps34 promotes autophagy and endocytosis but not TOR signaling in Drosophila . *Journal of Cell Biology*, *181*(4), 655–666. https://doi.org/10.1083/jcb.200712051

Justice, R. W., Zilian, O., Woods, D. F., Noll, M., & Bryant, P. J. (1995). The Drosophila tumor suppressor gene warts encodes a homolog of human myotonic dystrophy kinase and is required for the control of cell shape and proliferation. *Genes and Development*, *9*(5), 534–546. https://doi.org/10.1101/gad.9.5.534

Kalamegham, R., Sturgill, D., Siegfried, E., & Oliver, B. (2007). Drosophila mojoless, a Retroposed GSK-3, Has Functionally Diverged to Acquire an Essential Role in Male Fertility. *Molecular Biology and Evolution*, *24*(3), 732–742. https://doi.org/10.1093/molbev/msl201

Kanoh, H., Tong, L.-L., Kuraishi, T., Suda, Y., Momiuchi, Y., Shishido, F., & Kurata, S. (2015). Genome-wide RNAi screening implicates the E3 ubiquitin ligase Sherpa in mediating innate immune signaling by Toll in &lt;em&gt;Drosophila&lt;/em&gt; adults. *Science Signaling*, *8*(400), ra107 LP-ra107. https://doi.org/10.1126/scisignal.2005971

Kaplan, N. A., Colosimo, P. F., Liu, X., & Tolwinski, N. S. (2011). Complex Interactions between GSK3 and aPKC in Drosophila Embryonic Epithelial Morphogenesis. *PLoS ONE*, *6*(4), e18616. https://doi.org/10.1371/journal.pone.0018616

Khuong, T. M., Habets, R. L. P., Slabbaert, J. R., & Verstreken, P. (2010). WASP is activated by phosphatidylinositol-4,5-bisphosphate to restrict synapse growth in a pathway parallel to bone morphogenetic protein signaling. *Proceedings of the National Academy of Sciences*, *107*(40), 17379 LP – 17384. https://doi.org/10.1073/pnas.1001794107

Kidd, D., & Raff, J. W. (1997). LK6, a short lived protein kinase in Drosophila that can associate with microtubules and centrosomes. *Journal of Cell Science*, *110*(2), 209 LP – 219. http://jcs.biologists.org/content/110/2/209.abstract

Kiger, A. A., Baum, B., Jones, S., Jones, M. R., Coulson, A., Echeverri, C., & Perrimon, N. (2003). A functional genomic analysis of cell morphology using RNA interference. *Journal of Biology*, *2*(4), 27. https://doi.org/10.1186/1475-4924-2-27

Kim, J., Bang, H., Ko, S., Jung, I., Hong, H., & Kim-Ha, J. (2008). Drosophila ia2 modulates secretion of insulin-like peptide. *Comparative Biochemistry and Physiology Part A: Molecular & Integrative Physiology*, *151*(2), 180–184. https://doi.org/https://doi.org/10.1016/j.cbpa.2008.06.020

Kim, M., Lee, J. H., Koh, H., Lee, S. Y., Jang, C., Chung, C. J., Sung, J. H., Blenis, J., & Chung, J. (2006). Inhibition of ERK-MAP kinase signaling by RSK during Drosophila development. *The EMBO Journal*, *25*(13), 3056–3067. <https://doi.org/10.1038/sj.emboj.7601180>

Klinedinst, S., Wang, X., Xiong, X., Haenfler, J. M., & Collins, C. A. (2013). Independent Pathways Downstream of the Wnd/DLK MAPKKK Regulate Synaptic Structure, Axonal Transport, and Injury Signaling. *The Journal of Neuroscience*, *33*(31), 12764 LP – 12778. https://doi.org/10.1523/JNEUROSCI.5160-12.2013

Kondo, S., & Perrimon, N. (2011). A Genome-Wide RNAi Screen Identifies Core Components of the G&lt;sub&gt;2&lt;/sub&gt;-M DNA Damage Checkpoint. *Science Signaling*, *4*(154), rs1 LP-rs1. https://doi.org/10.1126/scisignal.2001350

Kunda, P., Pelling, A. E., Liu, T., & Baum, B. (2008). Moesin Controls Cortical Rigidity, Cell Rounding, and Spindle Morphogenesis during Mitosis. *Current Biology*, *18*(2), 91–101. https://doi.org/10.1016/j.cub.2007.12.051

Kuranaga, E., Kanuka, H., Tonoki, A., Takemoto, K., Tomioka, T., Kobayashi, M., Hayashi, S., & Miura, M. (2006). Drosophila IKK-Related Kinase Regulates Nonapoptotic Function of Caspases via Degradation of IAPs. *Cell*, *126*(3), 583–596. https://doi.org/10.1016/j.cell.2006.05.048

Kushimura, Y., Azuma, Y., Mizuta, I., Muraoka, Y., Kyotani, A., Yoshida, H., Tokuda, T., Mizuno, T., & Yamaguchi, M. (2018). Loss-of-function mutation in Hippo suppressed enlargement of lysosomes and neurodegeneration caused by dFIG4 knockdown. *NeuroReport*, *29*(10). https://journals.lww.com/neuroreport/Fulltext/2018/07010/Loss_of_function_mutation_in_Hippo_suppressed.12.aspx

Larochelle, S., Pandur, J., Fisher, R. P., Salz, H. K., & Suter, B. (1998). Cdk7 is essential for mitosis and for in vivo Cdk-activating kinase activity. *Genes & Development* , *12*(3), 370–381. http://genesdev.cshlp.org/content/12/3/370.abstract

Lee, H. K., Cording, A., Vielmetter, J., & Zinn, K. (2013). Interactions between a receptor tyrosine phosphatase and a cell surface ligand regulate axon guidance and glial-neuronal communication. *Neuron*, *78*(5), 813–826. https://doi.org/10.1016/j.neuron.2013.04.001

Lee, J. H., Koh, H., Kim, M., Kim, Y., Lee, S. Y., Karess, R. E., Lee, S. H., Shong, M., Kim, J. M., Kim, J., & Chung, J. (2007). Energy-dependent regulation of cell structure by AMP-activated protein kinase. *Nature*, *447*(7147), 1017–1020. https://doi.org/10.1038/nature05828

Lee, P. T., Lin, G., Lin, W. W., Diao, F., White, B. H., & Bellen, H. J. (2018). A kinase-dependent feedforward loop affects CREBB stability and long term memory formation. *ELife*, *7*. https://doi.org/10.7554/eLife.33007

Lee, S. J., & Montell, C. (2004). Light-dependent translocation of visual arrestin regulated by the NINAC myosin III. *Neuron*, *43*(1), 95–103. https://doi.org/10.1016/j.neuron.2004.06.014

Lee, S.-J., Feldman, R., & O’Farrell, P. H. (2008). An RNA Interference Screen Identifies a Novel Regulator of Target of Rapamycin That Mediates Hypoxia Suppression of Translation in Drosophila S2 Cells. *Molecular Biology of the Cell*, *19*(10), 4051–4061. https://doi.org/10.1091/mbc.e08-03-0265

Lee, W., Swarup, S., Chen, J., Ishitani, T., & Verheyen, E. M. (2009). Homeodomain-interacting protein kinases (Hipks) promote Wnt/Wg signaling through stabilization of β-catenin/Arm and stimulation of target gene expression. *Development*, *136*(2), 241–251. https://doi.org/10.1242/dev.025460

Lee, W. J., Kim, S. H., Kim, Y. S., Han, S. J., Park, K. S., Ryu, J. H., Hur, M. W., & Choi, K. Y. (2000). Inhibition of mitogen-activated protein kinase by a Drosophila dual-specific phosphatase. *Biochemical Journal*, *349*(3), 821–828. https://doi.org/10.1042/bj3490821

Li, M. Y., Lai, P. L., Chou, Y. T., Chi, A. P., Mi, Y. Z., Khoo, K. H., Chang, G. D., Wu, C. W., Meng, T. C., & Chen, G. C. (2015). Protein tyrosine phosphatase PTPN3 inhibits lung cancer cell proliferation and migration by promoting EGFR endocytic degradation. *Oncogene*, *34*(29), 3791–3803. https://doi.org/10.1038/onc.2014.312

Lindmo, K., Brech, A., Finley, K. D., Gaumer, S., Contamine, D., Rusten, T. E., & Stenmark, H. (2008). The PI 3-kinase regulator Vps15 is required for autophagic clearance of protein aggregates. *Autophagy*, *4*(4), 500–506. https://doi.org/10.4161/auto.5829

Liu, Z., Matsuoka, S., Enoki, A., Yamamoto, T., Furukawa, K., Yamasaki, Y., Nishida, Y., & Sugiyama, S. (2011). Negative modulation of bone morphogenetic protein signaling by Dullard during wing vein formation in Drosophila. *Development, Growth & Differentiation*, *53*(6), 822–841. https://doi.org/https://doi.org/10.1111/j.1440-169X.2011.01289.x

Livneh, E., Glazer, L., Segal, D., Schlessinger, J., & Shilo, B. Z. (1985). The Drosophila EGF receptor gene homolog: Conservation of both hormone binding and kinase domains. *Cell*, *40*(3), 599–607. https://doi.org/10.1016/0092-8674(85)90208-9

Logarinho, E., Bousbaa, H., Dias, J. M., Lopes, C., Amorim, I., Antunes-Martins, A., & Sunkel, C. E. (2004). Different spindle checkpoint proteins monitor microtubule attachment and tension at kinetochores in Drosophila cells. *Journal of Cell Science*, *117*(9), 1757–1771. https://doi.org/10.1242/jcs.01033

Loh, B. J., Cullen, C. F., Vogt, N., & Ohkura, H. (2012). The conserved kinase SRPK regulates karyosome formation and spindle microtubule assembly in Drosophila oocytes. *Journal of Cell Science*, *125*(19), 4457–4462. https://doi.org/10.1242/jcs.107979

Long, A. A., Mahapatra, C. T., Woodruff, E. A., Rohrbough, J., Leung, H. T., Shino, S., An, L., Doerge, R. W., Metzstein, M. M., Pak, W. L., & Broadie, K. (2010). The nonsense-mediated decay pathway maintains synapse architecture and synaptic vesicle cycle efficacy. *Journal of Cell Science*, *123*(19), 3303–3315. https://doi.org/10.1242/jcs.069468

Luebbering, N., Charlton-Perkins, M., Kumar, J. P., Rollmann, S. M., Cook, T., & Cleghon, V. (2013). Drosophila Dyrk2 Plays a Role in the Development of the Visual System. *PLoS ONE*, *8*(10), e76775. https://doi.org/10.1371/journal.pone.0076775

Luo, L., Lee, T., Tsai, L., Tang, G., Jan, L. Y., & Jan, Y. N. (1997). Genghis Khan (Gek) as a putative effector for Drosophila Cdc42 and regulator of actin polymerization. *Proceedings of the National Academy of Sciences of the United States of America*, *94*(24), 12963–12968. https://doi.org/10.1073/pnas.94.24.12963

Macagno, J. P., Diaz Vera, J., Yu, Y., MacPherson, I., Sandilands, E., Palmer, R., Norman, J. C., Frame, M., & Vidal, M. (2014). FAK Acts as a Suppressor of RTK-MAP Kinase Signalling in Drosophila melanogaster Epithelia and Human Cancer Cells. *PLoS Genetics*, *10*(3), e1004262. https://doi.org/10.1371/journal.pgen.1004262

Maier, D., Hausser, A., Nagel, A. C., Link, G., Kugler, S. J., Wech, I., Pfizenmaier, K., & Preiss, A. (2006). Drosophila protein kinase D is broadly expressed and a fraction localizes to the Golgi compartment. *Gene Expression Patterns*, *6*(8), 849–856. https://doi.org/10.1016/j.modgep.2006.03.007

Maier, D., Nagel, A. C., Gloc, H., Hausser, A., Kugler, S. J., Wech, I., & Preiss, A. (2007). Protein Kinase D regulates several aspects of development in Drosophila melanogaster. *BMC Developmental Biology*, *7*(1), 74. https://doi.org/10.1186/1471-213X-7-74

Malzer, E., Daly, M. L., Moloney, A., Sendall, T. J., Thomas, S. E., Ryder, E., Ryoo, H. D., Crowther, D. C., Lomas, D. A., & Marciniak, S. J. (2010). Impaired tissue growth is mediated by checkpoint kinase 1 (CHK1) in the integrated stress response. *Journal of Cell Science*, *123*(17), 2892–2900. https://doi.org/10.1242/jcs.070078

Marques, G., Haerry, T. E., Crotty, M. L., Xue, M., Zhang, B., & O’Connor, M. B. (2003). Retrograde Gbb signaling through the Bmp type 2 receptor wishful thinking regulates systemic FMRFa expression in Drosophila. *Development*, *130*(22), 5457–5470. https://doi.org/10.1242/dev.00772

Martín-Blanco, E., Gampel, A., Ring, J., Virdee, K., Kirov, N., Tolkovsky, A. M., & Martinez-Arias, A. (1998). puckered encodes a phosphatase that mediates a feedback loop regulating JNK activity during dorsal closure in Drosophila. *Genes Dev.*, *12*, 557–570. http://genesdev.cshlp.org/content/12/4/557

McElwain, M. A., Ko, D. C., Gordon, M. D., Fyrst, H., Saba, J. D., & Nusse, R. (2011). A Suppressor/Enhancer Screen in Drosophila Reveals a Role for Wnt-Mediated Lipid Metabolism in Primordial Germ Cell Migration. *PLoS ONE*, *6*(11), e26993. https://doi.org/10.1371/journal.pone.0026993

Mentzel, B., & Raabe, T. (2005). Phylogenetic and structural analysis of the Drosophila melanogaster p21-activated kinase DmPAK3. *Gene*, *349*, 25–33. https://doi.org/https://doi.org/10.1016/j.gene.2004.12.030

Menzel, N., Melzer, J., Waschke, J., Lenz, C., Wecklein, H., Lochnit, G., Drenckhahn, D., & Raabe, T. (2008). The Drosophila p21-activated kinase Mbt modulates DE-cadherin-mediated cell adhesion by phosphorylation of Armadillo. *Biochemical Journal*, *416*(2), 231–241. https://doi.org/10.1042/BJ20080465

Meyer, C. A., Jacobs, H. W., Datar, S. A., Du, W., Edgar, B. A., & Lehner, C. F. (2000). Drosophila Cdk4 is required for normal growth and is dispensable for cell cycle progression. *EMBO Journal*, *19*(17), 4533–4542. https://doi.org/10.1093/emboj/19.17.4533

Miura, G. I., Roignant, J. Y., Wassef, M., & Treisman, J. E. (2008). Myopic acts in the endocytic pathway to enhance signaling by the Drosophila EGF receptor. *Development*, *135*(11), 1913–1922. https://doi.org/10.1242/dev.017202

Mizuno, T., Amano, M., Kaibuchi, K., & Nishida, Y. (1999). Identification and characterization of Drosophila homolog of Rho-kinase. *Gene*, *238*(2), 437—444. https://doi.org/10.1016/s0378-1119(99)00351-0

Mizuno, T., Amano, M., Kaibuchi, K., & Nishida, Y. (1999). Identification and characterization of Drosophila homolog of Rho-kinase. *Gene*, *238*(2), 437–444. https://doi.org/10.1016/S0378-1119(99)00351-0

Molnar, C., Holguin, H., Mayor, F., Ruiz-Gomez, A., & De Celis, J. F. (2007). The G protein-coupled receptor regulatory kinase GPRK2 participates in Hedgehog signaling in Drosophila. *Proceedings of the National Academy of Sciences of the United States of America*. <https://doi.org/10.1073/pnas.0702374104>

Munch-Petersen, B., Knecht, W., Lenz, C., Søndergaard, L., & Piškur, J. (2000). Functional Expression of a Multisubstrate Deoxyribonucleoside Kinase from Drosophila melanogaster and  Its C-terminal Deletion Mutants . *Journal of Biological Chemistry* , *275*(9), 6673–6679. https://doi.org/10.1074/jbc.275.9.6673

Mummery-Widmer J.L., Masakazu Yamazaki, Thomas Stoeger, MariaNovatchkova, Sheetal Bhalerao, Doris Chen, Georg Dietzl, Barry J. Dickson, & Juergen A. Knoblich. (2009). *Genome-wide analysis of Notch signalling in Drosophila by transgenic RNAi Europe PMC Funders Group*. *458*(7241), 987–992. https://doi.org/10.1038/nature07936

Munneke, L. R., & Collier, G. E. (1988). Cytoplasmic and mitochondrial arginine kinases inDrosophila: Evidence for a single gene. *Biochemical Genetics*, *26*(1), 131–141. https://doi.org/10.1007/BF00555494

Murray, M. J., Davidson, C. M., Hayward, N. M., & Brand, A. H. (2006). The Fes/Fer non-receptor tyrosine kinase cooperates with Src42A to regulate dorsal closure in Drosophila. *Development*, *133*(16), 3063–3073. https://doi.org/10.1242/dev.02467

Nagoshi, E., Sugino, K., Kula, E., Okazaki, E., Tachibana, T., Nelson, S., & Rosbash, M. (2010). Dissecting differential gene expression within the circadian neuronal circuit of Drosophila. *Nature Neuroscience*, *13*(1), 60–68. <https://doi.org/10.1038/nn.2451>

Nam, S. C., & Choi, K. W. (2003). Interaction of Par-6 and Crumbs complexes is essential for photoreceptor morphogenesis in Drosophila. *Development*, *130*(18), 4363–4372. <https://doi.org/10.1242/dev.00648>

Nellen, D., Affolter, M., & Basler, K. (1994). Receptor serine/threonine kinases implicated in the control of Drosophila body pattern by decapentaplegic. *Cell*, *78*(2), 225–237. https://doi.org/10.1016/0092-8674(94)90293-3

Neubueser, D., & Hipfner, D. R. (2010). Overlapping Roles of Drosophila Drak and Rok Kinases in Epithelial Tissue Morphogenesis. *Molecular Biology of the Cell*, *21*(16), 2869–2879. https://doi.org/10.1091/mbc.e10-04-0328

Nil, Z., Millán, R. H., Gerbich, T., Leal, P., Yu, Z., Saraf, A., Sardiu, M., Lange, J. J., Yi, K., Unruh, J., Slaughter, B., & Si, K. (2019). Amyloid-like Assembly Activates a Phosphatase in the Developing Drosophila Embryo. *Cell*, *178*(6), 1403-1420.e21. <https://doi.org/10.1016/j.cell.2019.08.019>

Oates, A. C., Bonkovsky, J. L., Irvine, D. V, Kelly, L. E., Thomas, J. B., & Wilks, A. F. (1998). Embryonic expression and activity of doughnut, a second RYK homolog in Drosophila. *Mechanisms of Development*, *78*(1), 165–169. https://doi.org/https://doi.org/10.1016/S0925-4773(98)00167-1

Oellers, N., & Hafen, E. (1996). Biochemical Characterization of RolledSem, an Activated Form of Drosophila Mitogen-activated Protein Kinase. *Journal of Biological Chemistry* , *271*(40), 24939–24944. https://doi.org/10.1074/jbc.271.40.24939

Ohlmeyer, J. T., & Kalderon, D. (1998). Hedgehog stimulates maturation of Cubitus interruptus into a labile transcriptional activator. *Nature*, *396*(6713), 749–753. https://doi.org/10.1038/25533

Ohshiro, T., Emori, Y., & Saigo, K. (2002). Ligand-dependent activation of breathless FGF receptor gene in Drosophila developing trachea. *Mechanisms of Development*, *114*(1–2), 3–11. https://doi.org/10.1016/S0925-4773(02)00042-4

Oishi, I., Sugiyama, S., Liu, Z.-J., Yamamura, H., Nishida, Y., & Minami, Y. (1997). A Novel Drosophila Receptor Tyrosine Kinase Expressed Specifically in the Nervous System: UNIQUE STRUCTURAL FEATURES AND IMPLICATION IN DEVELOPMENTAL SIGNALING . *Journal of Biological Chemistry* , *272*(18), 11916–11923. https://doi.org/10.1074/jbc.272.18.11916

Park, J., Lee, N., Kavoussi, A., Seo, J. T., Kim, C. H., & Moon, S. J. (2015). Ciliary Phosphoinositide Regulates Ciliary Protein Trafficking in Drosophila. *Cell Reports*, *13*(12), 2808–2816. https://doi.org/10.1016/j.celrep.2015.12.009

Pascual, A., Chaminade, M., & Préat, T. (2005). Ethanolamine kinase controls neuroblast divisions in Drosophila mushroom bodies. *Developmental Biology*, *280*(1), 177–186. https://doi.org/10.1016/j.ydbio.2005.01.017

Patel, U., Davies, S. A., & Myat, M. M. (2012). Receptor-type guanylyl cyclase Gyc76C is required for development of the &lt;em&gt;Drosophila&lt;/em&gt; embryonic somatic muscle. *Biology Open*, *1*(6), 507 LP – 515. https://doi.org/10.1242/bio.2012943

Penton, A., Chen, Y., Staehling-Hampton, K., Wrana, J. L., Attisano, L., Szidonya, J., Cassill, J. A., Massagué, J., & Hoffmann, F. M. (1994). Identification of two bone morphogenetic protein type I receptors in Drosophila and evidence that Brk25D is a decapentaplegic receptor. *Cell*, *78*(2), 239–250. https://doi.org/10.1016/0092-8674(94)90294-1

Peradziryi, H., Kaplan, N. A., Podleschny, M., Liu, X., Wehner, P., Borchers, A., & Tolwinski, N. S. (2011). PTK7/Otk interacts with Wnts and inhibits canonical Wnt signalling. *The EMBO Journal*, *30*(18), 3729–3740. https://doi.org/https://doi.org/10.1038/emboj.2011.236

Perkins, A. D., & Tanentzapf, G. (2014). An Ongoing Role for Structural Sarcomeric Components in Maintaining Drosophila melanogaster Muscle Function and Structure. *PLoS ONE*, *9*(6), e99362. https://doi.org/10.1371/journal.pone.0099362

Petruk, S., Sedkov, Y., Smith, S. T., Krajewski, W., Nakamura, T., Canaani, E., Croce, C. M., & Mazo, A. B. T.-M. in E. (2003). Purification and Biochemical Properties of the Drosophila TAC1 Complex. In *Chromatin and Chromatin Remodeling Enzymes, Part C* (Vol. 377, pp. 255–266). Academic Press. https://doi.org/https://doi.org/10.1016/S0076-6879(03)77015-3

Plongthongkum, N., Kullawong, N., Panyim, S., & Tirasophon, W. (2007). Ire1 regulated XBP1 mRNA splicing is essential for the unfolded protein response (UPR) in Drosophila melanogaster. *Biochemical and Biophysical Research Communications*, *354*(3), 789–794. https://doi.org/https://doi.org/10.1016/j.bbrc.2007.01.056

Poernbacher, I., Baumgartner, R., Marada, S. K., Edwards, K., & Stocker, H. (2012). Drosophila Pez acts in hippo signaling to restrict intestinal stem cell proliferation. *Current Biology*, *22*(5), 389–396. https://doi.org/10.1016/j.cub.2012.01.019

Poon, C. L. C., Lin, J. I., Zhang, X., & Harvey, K. F. (2011). The sterile 20-like kinase Tao-1 controls tissue growth by regulating the Salvador-Warts-Hippo pathway. *Developmental Cell*, *21*(5), 896–906. https://doi.org/10.1016/j.devcel.2011.09.012

Prakash, S., McLendon, H. M., Dubreuil, C. I., Ghose, A., Hwa, J., Dennehy, K. A., Tomalty, K. M. H., Clark, K. L., Van Vactor, D., & Clandinin, T. R. (2009). Complex interactions amongst N-cadherin, DLAR, and Liprin-α regulate Drosophila photoreceptor axon targeting. *Developmental Biology*, *336*(1), 10–19. https://doi.org/https://doi.org/10.1016/j.ydbio.2009.09.016

Price, D. M., Jin, Z., Rabinovitch, S., & Campbell, S. D. (2002). Ectopic expression of the drosophila Cdk1 inhibitory kinases, Wee1 and Myt1, interferes with the second mitotic wave and disrupts pattern formation during eye development. *Genetics*, *161*(2), 721–731. https://www.ncbi.nlm.nih.gov/pmc/articles/pmid/12072468/?tool=EBI

Price, M. A., & Kalderon, D. (2002). Proteolysis of the Hedgehog signaling effector Cubitus interruptus requires phosphorylation by Glycogen Synthase Kinase 3 and Casein Kinase 1. *Cell*, *108*(6), 823–835. https://doi.org/10.1016/S0092-8674(02)00664-5

Prigent, C., Glover, D. M., & Giet, R. (2005). Drosophila Nek2 protein kinase knockdown leads to centrosome maturation defects while overexpression causes centrosome fragmentation and cytokinesis failure. *Experimental Cell Research*, *303*(1), 1–13. <https://doi.org/https://doi.org/10.1016/j.yexcr.2004.04.052>

Raghu, P., Usher, K., Jonas, S., Chyb, S., Polyanovsky, A., & Hardie, R. C. (2000). Constitutive activity of the light-sensitive channels TRP and TRPL in the Drosophila diacylglycerol kinase mutant, rdgA. *Neuron*, *26*(1), 169–179. https://doi.org/10.1016/S0896-6273(00)81147-2

Read, R. D., Bach, E. A., & Cagan, R. L. (2004). Drosophila C-Terminal Src Kinase Negatively Regulates Organ Growth and Cell Proliferation through Inhibition of the Src, Jun N-Terminal Kinase, and STAT Pathways. *Molecular and Cellular Biology*, *24*(15), 6676–6689. https://doi.org/10.1128/mcb.24.15.6676-6689.2004

Read, R. D., Fenton, T. R., Gomez, G. G., Wykosky, J., Vandenberg, S. R., Babic, I., Iwanami, A., Yang, H., Cavenee, W. K., Mischel, P. S., Furnari, F. B., & Thomas, J. B. (2013). A Kinome-Wide RNAi Screen in Drosophila Glia Reveals That the RIO Kinases Mediate Cell Proliferation and Survival through TORC2-Akt Signaling in Glioblastoma. *PLoS Genetics*, *9*(2), e1003253. https://doi.org/10.1371/journal.pgen.1003253

Renault, A. D., Kunwar, P. S., & Lehmann, R. (2010). Lipid phosphate phosphatase activity regulates dispersal and bilateral sorting of embryonic germ cells in Drosophila. *Development*, *137*(11), 1815 LP – 1823. https://doi.org/10.1242/dev.046110

Resnik-Docampo, M., & de Celis, J. F. (2011). MAP4K3 is a component of the TORC1 signalling complex that modulates cell growth and viability in Drosophila melanogaster. *PLoS ONE*. https://doi.org/10.1371/journal.pone.0014528

Rimkus, S. A., Katzenberger, R. J., Trinh, A. T., Dodson, G. E., Tibbetts, R. S., & Wassarman, D. A. (2008). Mutations in String/CDC25 inhibit cell cycle re-entry and neurodegeneration in a Drosophila model of Ataxia telangiectasia. *Genes and Development*, *22*(9), 1205–1220. https://doi.org/10.1101/gad.1639608

Robbins, D. J., Nybakken, K. E., Kobayashi, R., Sisson, J. C., Bishop, J. M., & Thérond, P. P. (1997). Hedgehog elicits signal transduction by means of a large complex containing the kinesin-related protein costal2. *Cell*, *90*(2), 225–234. https://doi.org/10.1016/S0092-8674(00)80331-1

Rogers, E. M., Hsiung, F., Rodrigues, A. B., & Moses, K. (2005). Slingshot cofilin phosphatase localization is regulated by Receptor Tyrosine Kinases and regulates cytoskeletal structure in the developing Drosophila eye. *Mechanisms of Development*, *122*(11), 1194–1205. https://doi.org/https://doi.org/10.1016/j.mod.2005.07.002

Ruberte, E., Marty, T., Nellen, D., Affolter, M., & Basler, K. (1995). An absolute requirement for both the type II and type I receptors, punt and thick veins, for Dpp signaling in vivo. *Cell*, *80*(6), 889–897. <https://doi.org/10.1016/0092-8674(95)90292-9>

Rusten, T. E., Vaccari, T., Lindmo, K., Rodahl, L. M. W., Nezis, I. P., Sem-Jacobsen, C., Wendler, F., Vincent, J. P., Brech, A., Bilder, D., & Stenmark, H. (2007). ESCRTs and Fab1 Regulate Distinct Steps of Autophagy. *Current Biology*, *17*(20), 1817–1825. https://doi.org/10.1016/j.cub.2007.09.032

Qi, H., Yao, C., Cai, W., Girton, J., Johansen, K. M., & Johansen, J. (2009). Asator, a tau-tubulin kinase homolog in Drosophila localizes to the mitotic spindle. *Developmental Dynamics*, *238*(12), 3248–3256. https://doi.org/https://doi.org/10.1002/dvdy.22150

Sakurai, M., Aoki, T., Yoshikawa, S., Santschi, L. A., Saito, H., Endo, K., Ishikawa, K., Kimura, K., Ito, K., Thomas, J. B., & Hama, C. (2009). Differentially Expressed Drl and Drl-2 Play Opposing Roles in Wnt5 Signaling during &lt;em&gt;Drosophila&lt;/em&gt; Olfactory System Development. *The Journal of Neuroscience*, *29*(15), 4972 LP – 4980. https://doi.org/10.1523/JNEUROSCI.2821-08.2009

Santhanam, A., Peng, W.-H., Yu, Y.-T., Sang, T.-K., Chen, G.-C., & Meng, T.-C. (2014). Ecdysone-Induced Receptor Tyrosine Phosphatase PTP52F Regulates &lt;span class=&quot;named-content genus-species&quot; id=&quot;named-content-1&quot;&gt;Drosophila&lt;/span&gt; Midgut Histolysis by Enhancement of Autophagy and Apoptosis. *Molecular and Cellular Biology*, *34*(9), 1594 LP – 1606. https://doi.org/10.1128/MCB.01391-13

Santoyo, J., Alcalde, J., Méndez, R., Pulido, D., & de Haro, C. (1997). Cloning and Characterization of a cDNA Encoding a  Protein Synthesis Initiation Factor-2α (eIF-2α) Kinase  fromDrosophila melanogaster : HOMOLOGY TO YEAST GCN2 PROTEIN KINASE. *Journal of Biological Chemistry* , *272*(19), 12544–12550. https://doi.org/10.1074/jbc.272.19.12544

Scanga, S. E., Ruel, L., Binari, R. C., Snow, B., Stambolic, V., Bouchard, D., Peters, M., Calvieri, B., Mak, T. W., Woodgett, J. R., & Manoukian, A. S. (2000). The conserved PI3′K/PTEN/Akt signaling pathway regulates both cell size and survival in Drosophila. *Oncogene*, *19*(35), 3971–3977. https://doi.org/10.1038/sj.onc.1203739

Schnorrer, F., Schönbauer, C., Langer, C. C. H., Dietzl, G., Novatchkova, M., Schernhuber, K., Fellner, M., Azaryan, A., Radolf, M., Stark, A., Keleman, K., & Dickson, B. J. (2010). Systematic genetic analysis of muscle morphogenesis and function in Drosophila. *Nature*, *464*(7286), 287–291. https://doi.org/10.1038/nature08799

Schoenherr, J. A., Drennan, J. M., Martinez, J. S., Chikka, M. R., Hall, M. C., Chang, H. C., & Clemens, J. C. (2012). Drosophila Activated Cdc42 Kinase Has an Anti-Apoptotic Function. *PLoS Genetics*, *8*(5), e1002725. https://doi.org/10.1371/journal.pgen.1002725

Scott, R. C., Juhász, G., & Neufeld, T. P. (2007). Direct Induction of Autophagy by Atg1 Inhibits Cell Growth and Induces Apoptotic Cell Death. *Current Biology*, *17*(1), 1–11. <https://doi.org/10.1016/j.cub.2006.10.053>

Seisenbacher, G., Hafen, E., & Stocker, H. (2011). Mk2-dependent p38b signalling protects drosophila hindgut enterocytes against jnk-induced apoptosis under chronic stress. *PLoS Genetics*, *7*(8), e1002168. https://doi.org/10.1371/journal.pgen.1002168

Sekine, Y., Takagahara, S., Hatanaka, R., Watanabe, T., Oguchi, H., Noguchi, T., Naguro, I., Kobayashi, K., Tsunoda, M., Funatsu, T., Nomura, H., Toyoda, T., Matsuki, N., Kuranaga, E., Miura, M., Takeda, K., & Ichijo, H. (2011). P38 mapks regulate the expression of genes in the dopamine synthesis pathway through phosphorylation of NR4A nuclear receptors. *Journal of Cell Science*, *124*(17), 3006–3016. https://doi.org/10.1242/jcs.085902

Sepp, K. J., Hong, P., Lizarraga, S. B., Liu, J. S., Mejia, L. A., Walsh, C. A., & Perrimon, N. (2008). Identification of Neural Outgrowth Genes using Genome-Wide RNAi. *PLoS Genetics*, *4*(7), e1000111. https://doi.org/10.1371/journal.pgen.1000111

Serysheva, E., Berhane, H., Grumolato, L., Demir, K., Balmer, S., Bodak, M., Boutros, M., Aaronson, S., Mlodzik, M., & Jenny, A. (2013). Wnk kinases are positive regulators of canonical Wnt/β-catenin signalling. *EMBO Reports*, *14*(8), 718–725. https://doi.org/https://doi.org/10.1038/embor.2013.88

Sesé, M., Corominas, M., Stocker, H., Heino, T. I., Hafen, E., & Serras, F. (2006). The Cdi/TESK1 kinase is required for Sevenless signaling and epithelial organization in the &lt;em&gt;Drosophila&lt;/em&gt; eye. *Journal of Cell Science*, *119*(24), 5047 LP – 5056. https://doi.org/10.1242/jcs.03294

Shindo, M., Wada, H., Kaido, M., Tateno, M., Aigaki, T., Tsuda, L., & Hayashi, S. (2008). Dual function of Src in the maintenance of adherens junctions during tracheal epithelial morphogenesis. *Development*, *135*(7), 1355 LP – 1364. https://doi.org/10.1242/dev.015982

Shishido, E., Ono, N., Kojima, T., & Saigo, K. (1997). Requirements of DFR1/Heartless, a mesoderm-specific Drosophila FGF-receptor, for the formation of heart, visceral and somatic muscles, and ensheathing of longitudinal axon tracts in CNS. *Development*, *124*(11), 2119–2128.

Sluss, H. K., Han, Z., Barrett, T., Davis, R. J., & Ip, Y. T. (1996). A JNK signal transduction pathway that mediates morphogenesis and an immune response in Drosophila. *Genes & Development* , *10*(21), 2745–2758. https://doi.org/10.1101/gad.10.21.2745

Smith, D. P., Ranganathan, R., Hardy, R. W., Marx, J., Tsuchida, T., & Zuker, C. S. (1991). Photoreceptor deactivation and retinal degeneration mediated by a photoreceptor-specific protein kinase C. *Science*, *254*(5037), 1478 LP – 1484. https://doi.org/10.1126/science.1962207

Soba, P., Han, C., Zheng, Y., Perea, D., Miguel-Aliaga, I., Jan, L. Y., & Jan, Y. N. (2015). The ret receptor regulates sensory neuron dendrite growth and integrin mediated adhesion. *ELife*, *2015*(4). https://doi.org/10.7554/eLife.05491

Song, Y., Eng, M., & Ghabrial, A. S. (2013). Focal Defects in Single-Celled Tubes Mutant for Cerebral Cavernous Malformation 3, GCKIII, or NSF2. *Developmental Cell*, *25*(5), 507–519. https://doi.org/10.1016/j.devcel.2013.05.002

Steele, F., & O’Tousa, J. E. (1990). Rhodopsin activation causes retinal degeneration in drosophila rdgC mutant. *Neuron*, *4*(6), 883–890. https://doi.org/10.1016/0896-6273(90)90141-2

Stephenson, R., Hosler, M. R., Gavande, N. S., Ghosh, A. K., & Weake, V. M. (2015). Characterization of a Drosophila ortholog of the Cdc7 kinase a role for Cdc7 in endoreplication independent of chiffon. *Journal of Biological Chemistry*, *290*(3), 1332–1347. https://doi.org/10.1074/jbc.M114.597948

Stern, B., Ried, G., Clegg, N. J., Grigliatti, T. A., & Lehner, C. F. (1993). Genetic analysis of the Drosophila cdc2 homolog. *Development*, *117*(1), 219 LP – 232. http://dev.biologists.org/content/117/1/219.abstract

Stowers, R. S., Garza, D., Rascle, A., & Hogness, D. S. (2000). The L63 Gene Is Necessary for the Ecdysone-Induced 63E Late Puff and Encodes CDK Proteins Required for Drosophila Development. *Developmental Biology*, *221*(1), 23–40. https://doi.org/https://doi.org/10.1006/dbio.2000.9685

Stronach, B., & Perrimon, N. (2002). Activation of the JNK pathway during dorsal closure in Drosophila requires the mixed lineage kinase, slipper. *Genes and Development*, *16*(3), 377–387. https://doi.org/10.1101/gad.953002

Su, Y. C., Treisman, J. E., & Skolnik, E. Y. (1998). The Drosophila Ste20-related kinase misshapen is required for embryonic dorsal closure and acts through a JNK MAPK module on an evolutionarily conserved signaling pathway. *Genes and Development*, *12*(15), 2371–2380. https://doi.org/10.1101/gad.12.15.2371

Sugiyama, S., Moritoh, S., Furukawa, Y., Mizuno, T., Lim, Y.-M., Tsuda, L., & Nishida, Y. (2007). Involvement of the Mitochondrial Protein Translocator Component Tim50 in Growth, Cell Proliferation and the Modulation of Respiration in Drosophila. *Genetics*, *176*(2), 927 LP – 936. https://doi.org/10.1534/genetics.107.072074

Sun, L., Yu, M. C., Kong, L., Zhuang, Z. H., Hu, J. H., & Ge, B. X. (2008). Molecular identification and functional characterization of a Drosophila dual-specificity phosphatase DMKP-4 which is involved in PGN-induced activation of the JNK pathway. *Cellular Signalling*, *20*(7), 1329–1337. https://doi.org/10.1016/j.cellsig.2008.03.003

Sun, M., Liu, L., Zeng, X., Xu, M., Liu, L., Fang, M., & Xie, W. (2009). Genetic interaction between Neurexin and CAKI/CMG is important for synaptic function in Drosophila neuromuscular junction. *Neuroscience Research*, *64*(4), 362–371. https://doi.org/10.1016/j.neures.2009.04.009

Swarup, S., Pradhan-Sundd, T., & Verheyen, E. M. (2015). Genome-wide identification of phospho-regulators of Wnt signaling in Drosophila. *Development (Cambridge)*, *142*(8), 1502–1515. https://doi.org/10.1242/dev.116715

Takatsu, Y., Nakamura, M., Stapleton, M., Danos, M. C., Matsumoto, K., O’Connor, M. B., Shibuya, H., & Ueno, N. (2000). TAK1 Participates in c-Jun N-Terminal Kinase Signaling during Drosophila Development. *Molecular and Cellular Biology*, *20*(9), 3015 LP – 3026. https://doi.org/10.1128/MCB.20.9.3015-3026.2000

Takeo, S., Tsuda, M., Akahori, S., Matsuo, T., & Aigaki, T. (2006). The Calcineurin Regulator Sra Plays an Essential Role in Female Meiosis in Drosophila. *Current Biology*, *16*(14), 1435–1440. https://doi.org/10.1016/j.cub.2006.05.058

Tateno, M., Nishida, Y., & Adachi-Yamada, T. (2000). Regulation of JNK by Src During &lt;em&gt;Drosophila&lt;/em&gt; Development. *Science*, *287*(5451), 324 LP – 327. https://doi.org/10.1126/science.287.5451.324

Therrien, M., Chang, H. C., Solomon, N. M., Karim, F. D., Wassarman, D. A., & Rubin, G. M. (1995). KSR, a novel protein kinase required for RAS signal transduction. *Cell*, *83*(6), 879–888. https://doi.org/10.1016/0092-8674(95)90204-X

Tombácz, I., Schauer, T., Juhász, I., Komonyi, O., & Boros, I. (2009). The RNA Pol II CTD phosphatase Fcp1 is essential for normal development in Drosophila melanogaster. *Gene*, *446*(2), 58–67. https://doi.org/https://doi.org/10.1016/j.gene.2009.07.012

Tomlinson, A., & Ready, D. F. (1987). Cell fate in the Drosophila ommatidium. *Developmental Biology*, *123*(1), 264–275. https://doi.org/https://doi.org/10.1016/0012-1606(87)90448-9

Tran, T. A., Kinch, L., Pena-Llopis, S., Kockel, L., Grishin, N., Jiang, H., & Brugarolas, J. (2013). Platelet-Derived Growth Factor/Vascular Endothelial Growth Factor Receptor Inactivation by Sunitinib Results in Tsc1/Tsc2-Dependent Inhibition of TORC1. *Molecular and Cellular Biology*, *33*(19), 3762–3779. https://doi.org/10.1128/mcb.01570-12

Tremmel, D. M., Resad, S., Little, C. J., & Wesley, C. S. (2013). Notch and PKC Are Involved in Formation of the Lateral Region of the Dorso-Ventral Axis in Drosophila Embryos. *PLoS ONE*, *8*(7), e67789. https://doi.org/10.1371/journal.pone.0067789

Tsuda, L., Inoue, Y. H., Yoo, M. A., Mizuno, M., Hata, M., Lim, Y. M., Adachi-Yamada, T., Ryo, H., Masamune, Y., & Nishida, Y. (1993). A protein kinase similar to MAP kinase activator acts downstream of the raf kinase in Drosophila. *Cell*, *72*(3), 407–414. https://doi.org/10.1016/0092-8674(93)90117-9

Udan, R. S., Kango-Singh, M., Nolo, R., Tao, C., & Halder, G. (2003). Hippo promotes proliferation arrest and apoptosis in the Salvador/Warts pathway. *Nature Cell Biology*, *5*(10), 914–920. https://doi.org/10.1038/ncb1050

Vereshchagina, N., Bennett, D., Szöőr, B., Kirchner, J., Gross, S., Vissi, E., White-Cooper, H., & Alphey, L. (2004). The Essential Role of PP1β in Drosophila Is to Regulate Nonmuscle Myosin. *Molecular Biology of the Cell*, *15*(10), 4395–4405. https://doi.org/10.1091/mbc.e04-02-0139

Verheyen, E. M., Mirkovic, I., MacLean, S. J., Langmann, C., Andrews, B. C., & MacKinnon, C. (2001). The tissue polarity gene nemo carries out multiple roles in patterning during Drosophila development. *Mechanisms of Development*, *101*(1), 119–132. https://doi.org/https://doi.org/10.1016/S0925-4773(00)00574-8

Verstreken, P., Koh, T. W., Schulze, K. L., Zhai, R. G., Hiesinger, P. R., Zhou, Y., Mehta, S. Q., Cao, Y., Roos, J., & Bellen, H. J. (2003). Synaptojanin is recruited by endophilin to promote synaptic vesicle uncoating. *Neuron*, *40*(4), 733–748. https://doi.org/10.1016/S0896-6273(03)00644-5

Von Stetina, J. R., Tranguch, S., Dey, S. K., Lee, L. A., Cha, B., & Drummond-Barbosa, D. (2008). α-Endosulfine is a conserved protein required for oocyte meiotic maturation in Drosophila. *Development*, *135*(22), 3697–3706. https://doi.org/10.1242/dev.025114

Watson, K. L., Chou, M. M., Blenis, J., Gelbart, W. M., & Erikson, R. L. (1996). A Drosophila gene structurally and functionally  homologous to the mammalian 70-kDa S6 kinase gene. *Proceedings of the National Academy of Sciences*, *93*(24), 13694–13698. https://doi.org/10.1073/PNAS.93.24.13694

Weber, U., Gault, W. J., Olguin, P., Serysheva, E., & Mlodzik, M. (2012). Novel Regulators of Planar Cell Polarity: A Genetic Analysis in &lt;em&gt;Drosophila&lt;/em&gt; *Genetics*, *191*(1), 145 LP – 162. https://doi.org/10.1534/genetics.111.137190

Weinkove, D., Neufeld, T. P., Twardzik, T., Waterfield, M. D., & Leevers, S. J. (1999). Regulation of imaginal disc cell size, cell number and organ size by Drosophila class I(A) phosphoinositide 3-kinase and its adaptor. *Current Biology*, *9*(18), 1019–1029. https://doi.org/10.1016/S0960-9822(99)80450-3

Werner-Allen, J. W., Lee, C.-J., Liu, P., Nicely, N. I., Wang, S., Greenleaf, A. L., & Zhou, P. (2011). cis-Proline-mediated Ser(P)5 Dephosphorylation by the RNA Polymerase II C-terminal Domain Phosphatase Ssu72. *Journal of Biological Chemistry* , *286*(7), 5717–5726. https://doi.org/10.1074/jbc.M110.197129

Wilson, C., Goberdhan, D. C., & Steller, H. (1993). Dror, a potential neurotrophic receptor gene, encodes a Drosophila homolog of the vertebrate Ror family of Trk-related receptor tyrosine kinases. *Proceedings of the National Academy of Sciences*, *90*(15), 7109–7113. https://doi.org/10.1073/PNAS.90.15.7109

Xu, J., Xin, S., & Du, W. (2001). *Drosophila* Chk2 is required for DNA damage-mediated cell cycle arrest and apoptosis. *FEBS Letters*, *508*(3), 394–398. https://doi.org/10.1016/S0014-5793(01)03103-9

Yamaguchi, T., Fernandez, R., & Roth, R. A. (1995). Comparison of the Signaling Abilities of the Drosophila and Human Insulin Receptors in Mammalian Cells. *Biochemistry*, *34*(15), 4962–4968. <https://doi.org/10.1021/bi00015a007>

Yamaguchi, S., Katagiri, S., Sekimizu, K., Natori, S., & Homma, K. J. (2005). Involvement of EDTP, an Egg-Derived Tyrosine Phosphatase, in the Early Development of Drosophila melanogaster. *The Journal of Biochemistry*, *138*(6), 721–728. https://doi.org/10.1093/jb/mvi176

Yan, Y., Denef, N., Tang, C., & Schüpbach, T. (2011). Drosophila PI4KIIIalpha is required in follicle cells for oocyte polarization and Hippo signaling. *Development*, *138*(9), 1697–1703. https://doi.org/10.1242/dev.059279

Yanagawa, S., Matsuda, Y., Lee, J.-S., Matsubayashi, H., Sese, S., Kadowaki, T., & Ishimoto, A. (2002). Casein kinase I phosphorylates the Armadillo protein and induces its degradation in Drosophila. *The EMBO Journal*, *21*(7), 1733–1742. https://doi.org/https://doi.org/10.1093/emboj/21.7.1733

Yang, M. Y., Wang, Z., MacPherson, M., Dow, J. A., & Kaiser, K. (2000). A novel Drosophila alkaline phosphatase specific to the ellipsoid body of the adult brain and the lower Malpighian (renal) tubule. *Genetics*, *154*(1), 285–297. https://pubmed.ncbi.nlm.nih.gov/10628988

Yang, Y., Gehrke, S., Imai, Y., Huang, Z., Ouyang, Y., Wang, J.-W., Yang, L., Beal, M. F., Vogel, H., & Lu, B. (2006). Mitochondrial pathology and muscle and dopaminergic neuron degeneration caused by inactivation of &lt;em&gt;Drosophila&lt;/em&gt; Pink1 is rescued by Parkin. *Proceedings of the National Academy of Sciences*, *103*(28), 10793 LP – 10798. https://doi.org/10.1073/pnas.0602493103

Yanicostas, C., Vincent, A., & Lepesant, J. A. (1989). Transcriptional and posttranscriptional regulation contributes to the sex-regulated expression of two sequence-related genes at the janus locus of Drosophila melanogaster. *Molecular and Cellular Biology*, *9*(6), 2526 LP – 2535. https://doi.org/10.1128/MCB.9.6.2526

Yao, Y., Wu, Y., Yin, C., Ozawa, R., Aigaki, T., Wouda, R. R., Noordermeer, J. N., Fradkin, L. G., & Hing, H. (2007). Antagonistic roles of Wnt5 and the Drl receptor in patterning the Drosophila antennal lobe. *Nature Neuroscience*, *10*(11), 1423–1432. https://doi.org/10.1038/nn1993

Yasbin, R., Sawicki, J., & MacIntyre, R. J. (1978). A developmental study of acid phosphatase-1 in Drosophila melanogaster. *Developmental Biology*, *63*(1), 35–46. https://doi.org/https://doi.org/10.1016/0012-1606(78)90111-2

Yoon, W., Hwang, S.-H., Lee, S.-H., & Chung, J. (2019). Drosophila ADCK1 is critical for maintaining mitochondrial structures and functions in the muscle. *PLOS Genetics*, *15*(5), e1008184. https://doi.org/10.1371/journal.pgen.1008184

Yoshida, M., Matsuda, H., Kubo, H., & Nishimura, T. (2016). Molecular characterization of Tps1 and Treh genes in Drosophila and their role in body water homeostasis. *Scientific Reports*, *6*(1), 30582. https://doi.org/10.1038/srep30582

Zacharogianni, M., Kondylis, V., Tang, Y., Farhan, H., Xanthakis, D., Fuchs, F., Boutros, M., & Rabouille, C. (2011). ERK7 is a negative regulator of protein secretion in response to amino-acid starvation by modulating Sec16 membrane association. *The EMBO Journal*, *30*(18), 3684–3700. https://doi.org/10.1038/emboj.2011.253

Zahedi, B., Shen, W., Xu, X., Chen, X., Mahey, M., & Harden, N. (2008). Leading edge-secreted Dpp cooperates with ACK-dependent signaling from the amnioserosa to regulate myosin levels during dorsal closure. *Developmental Dynamics*, *237*(10), 2936–2946. https://doi.org/10.1002/dvdy.21722

Zervas, C. G., Gregory, S. L., & Brown, N. H. (2001). Drosophila integrin-linked kinase is required at sites of integrin adhesion to link the cytoskeleton to the plasma membrane. *Journal of Cell Biology*, *152*(5), 1007–1018. https://doi.org/10.1083/jcb.152.5.1007

Zhang, L., Jia, J., Wang, B., Amanai, K., Wharton, K. A., & Jiang, J. (2006). Regulation of wingless signaling by the CKI family in Drosophila limb development. *Developmental Biology*, *299*(1), 221–237. https://doi.org/https://doi.org/10.1016/j.ydbio.2006.07.025

Zhang, W., Yang, J., Liu, Y., Chen, X., Yu, T., Jia, J., & Liu, C. (2009). PR55α, a regulatory subunit of PP2A, specifically regulates PP2A-mediated β-catenin dephosphorylation. *Journal of Biological Chemistry*, *284*(34), 22649–22656. https://doi.org/10.1074/jbc.M109.013698

Zhao, G., Wu, Y., Du, L., Li, W., Xiong, Y., Yao, A., Wang, Q., & Zhang, Y. Q. (2015). Drosophila S6 Kinase Like Inhibits Neuromuscular Junction Growth by Downregulating the BMP Receptor Thickveins. *PLOS Genetics*, *11*(3), e1004984. https://doi.org/10.1371/journal.pgen.1004984
