# Supplementary material for: Functional requirements of protein kinases and phosphatases in the development of the Drosophila melanogaster wing
Source: G3 (Bethesda). 2021 Oct 2;11(12):jkab348. doi: 10.1093/g3journal/jkab348 (PMC8664455; doi:10.1093/g3journal/jkab348)
Supplement: jkab348_Supplementary_Data [file jkab348_supplementary_data.zip › GENETICS-G3-2021-402588-s02.docx]

**Supplementary Figure 1**

Wing phenotypes resulting from knock-down of some previously characterized protein kinases. All combinations are in *UAS-Dicer2/+; nub-Gal4/UAS-RNAi* except (J) which is *UAS-Dicer2/+; sal^EPv^-Gal4/UAS-RNAi*. Each gene is named at the bottom of each picture.

**Supplementary Figure 2**

Wing phenotypes resulting from knock-down of protein kinases of the CMGC and STE groups. All combinations are in *UAS-Dicer2/+; nub-Gal4/UAS-RNAi* unless indicated by “sal” at the bottom of wing pictures (*UAS-Dicer2/+; sal^EPv^-Gal4/UAS-RNAi*). Each gene is named at the bottom of each picture. Genes not tested are written in red and genes which knock-down results in a wild type wing are written in black.

**Supplementary Figure 3**

Wing phenotypes resulting from knock-down of protein kinases of the APK, OPK and CKI groups. All combinations are in *UAS-Dicer2/+; nub-Gal4/UAS-RNAi* unless indicated by “sal” at the bottom of wing pictures (*UAS-Dicer2/+; sal^EPv^-Gal4/UAS-RNAi*). Each gene is named at the bottom of each picture. Genes not tested are written in red and genes which knock-down results in a wild type wing are written in black.

**Supplementary Figure 4**

Wing phenotypes resulting from knock-down of protein kinases of the AGC and CAMK groups. All combinations are in *UAS-Dicer2/+; nub-Gal4/UAS-RNAi* unless indicated by “sal” at the bottom of wing pictures (*UAS-Dicer2/+; sal^EPv^-Gal4/UAS-RNAi*). Each gene is named at the bottom of each picture. Genes not tested are written in red and genes which knock-down results in a wild type wing are written in black.

**Supplementary Figure 5**

Wing phenotypes resulting from knock-down of protein kinases of the TLK, TK and Lipid kinase groups. All combinations are in *UAS-Dicer2/+; nub-Gal4/UAS-RNAi* unless indicated by “sal” at the bottom of wing pictures (*UAS-Dicer2/+; sal^EPv^-Gal4/UAS-RNAi*). Each gene is named at the bottom of each picture. Genes not tested are written in red and genes which knock-down results in a wild type wing are written in black.

**Supplementary Figure 6**

Wing phenotypes resulting from knock-down of protein phosphatases of the Lipid phosphatase, IPP and PPP groups. All combinations are in *UAS-Dicer2/+; nub-Gal4/UAS-RNAi* unless indicated by “sal” at the bottom of wing pictures (*UAS-Dicer2/+; sal^EPv^-Gal4/UAS-RNAi*). Each gene is named at the bottom of each picture. Genes not tested are written in red and genes which knock-down results in a wild type wing are written in black.

**Supplementary Figure 7**

Wing phenotypes resulting from knock-down of protein phosphatases of the PPM, cytosolic PTP, receptor PTP and DUSP groups. All combinations are in *UAS-Dicer2/+; nub-Gal4/UAS-RNAi* unless indicated by “sal” at the bottom of wing pictures (*UAS-Dicer2/+; sal^EPv^-Gal4/UAS-RNAi*). Each gene is named at the bottom of each picture. Genes not tested are written in red and genes which knock-down results in a wild type wing are written in black.

**Supplementary Table 1**

Classification, wing phenotype in combination of UAS-RNAi (RNAi) with nub-Gal4 (nub>dicer) and salEPv-Gal4 (sal>dicer), human orthologs (H Ort), molecular function (MF), main bibliographic reference (REF), functional class (MC) and expression in the wing disc (EXP) of *Drosophila* kinases and phosphatases.

MC: sugar and lipid metabolism (MET), signaling (SIG), cytoskeleton organization (CYT), protein transport across membranes (PTR), cell adhesion (CA), RNA biology (RNA), DNA biology (DNA), protein modifications (PRO), cell division (DIV), immunological responses (IMM), solute transport (TRA). The abbreviations used to describe each phenotype are described in the main text. Tabs “Kinases b” and “Phosphatases b” contain the same information but in a different format that might be useful to search for individual phenotypes. Red cells correspond to genes not analyzed, and red lettering to genes for which the UAS-RNAi strain we used gives a wild type phenotype but were defined as showing a “wing phenotype” with other UAS-RNAi lines (Swarup *et al*. 2015).

**Supplementary Table 2**

Modifications of EGFR and ERK phenotypes by knock-down of kinases and phosphatases. No modification (=), phenotypic enhancement of the background phenotype (E) and phenotypic suppression of the background phenotype (S).

**Supplementary Table 3**

This Table correspond to Table 1 of the main manuscript in a xlsx format.

**Supplementary information**

List of references cited in Table 1 and Supplementary Table 1.
